# A toll-like receptor 9 antagonist restores below-level glial glutamate transporter expression in the dorsal horn following spinal cord injury

**DOI:** 10.1038/s41598-018-26915-2

**Published:** 2018-06-07

**Authors:** Alexandra Pallottie, Ayomi Ratnayake, Li Ni, Cigdem Acioglu, Lun Li, Ersilia Mirabelli, Robert F. Heary, Stella Elkabes

**Affiliations:** 1 The Reynolds Family Spine Laboratory, New Jersey Medical School, Department of Neurological Surgery, Rutgers, The State University of New Jersey, Newark, NJ 07103 USA; 2 The School of Graduate Studies, New Jersey Medical School, Rutgers, The State University of New Jersey, Newark, NJ 07103 USA

## Abstract

Spinal cord (SC) trauma elicits pathological changes at the primary lesion and in regions distant from the injury epicenter. Therapeutic agents that target mechanisms at the injury site are likely to exert additional effects in these remote regions. We previously reported that a toll-like receptor 9 (TLR9) antagonist, oligodeoxynucleotide 2088 (ODN 2088), improves functional deficits and modulates the milieu at the epicenter in mice sustaining a mid-thoracic contusion. The present investigations use the same paradigm to assess ODN 2088-elicited alterations in the lumbar dorsal horn (LDH), a region remote from the injury site where SCI-induced molecular alterations have been well defined. We report that ODN 2088 counteracts the SCI-elicited decrease in glial glutamate aspartate transporter (GLAST) and glutamate transporter 1 (GLT1) levels, whereas the levels of the neuronal glutamate transporter excitatory amino acid carrier 1 (EAAC1) and astroglial GABA transporter 3 (GAT3) were unaffected. The restoration of GLAST and GLT1 was neither paralleled by a global effect on astrocyte and microglia activation nor by changes in the expression of cytokines and growth factors reported to regulate these transporters. We conclude that the effects of intrathecal ODN 2088 treatment extend to loci beyond the epicenter by selectively targeting glial glutamate transporters.

## Introduction

Traumatic spinal cord injury (SCI) triggers a cascade of molecular and cellular events at the injury epicenter, including the infiltration of immune system cells, which induces an inflammatory reaction that exacerbates tissue damage caused by the initial mechanical trauma^1,2^. Disruption of the axonal tracts that connect the brain and the spinal cord (SC) also elicits effects in SC regions that are remote from the epicenter, such as the lumbar dorsal horn (LDH)^3–5^. The SCI-elicited changes in the DH of remote regions have been best characterized in the context of pain mechanisms, since the second-order sensory neurons which convey nociceptive information to the brain are localized to the DH. Glutamate transporters and receptors assume a central role in the hyperexcitation of these neurons following SCI.

Synaptic glutamate levels are partly dependent on the extent of its release as well as the active uptake by surrounding cells through high affinity glutamate transporters^6^. Glutamate-aspartate transporter (GLAST)^7^, glutamate transporter 1 (GLT1)^8^, and excitatory amino acid carrier 1 (EAAC1)^9^ are members of the solute carrier 1 (SLC1) family of transmembrane proteins and are expressed throughout the SC; however, they are most predominantly expressed in the DH^6^. GLT1 accounts for approximately 40% of all spinal glutamate transporters and it is most abundant in the LDH^6^. Previous studies indicate that reductions in SC GLAST and GLT1 expression result in decreased glutamate uptake^10–13^ and lead to hyperexcitation of neurons via the overstimulation of glutamate receptors^14^. Whereas EAAC1 is primarily found in neurons^15,16^, both GLAST and GLT1 are predominantly expressed in astrocytes^16,17^.

Astrocytes, microglia, and neurons express toll-like receptors (TLRs)^18–21^. TLRs are well known for their ability to bind components of pathogens which are referred to as pathogen-associated molecular patterns (PAMPs)^22^. In addition to their role in initiating innate immunity in response to infection, TLRs bind endogenous ligands known as danger associated molecular patterns (DAMPs) that are released from damaged or necrotic cells^23–25^. As a result, TLRs have been implicated as mediators of sterile inflammation associated with central nervous system (CNS) injury^20,21,26^. Thirteen TLRs have been identified in mice. The focus of the present study is TLR9 which is found in both humans and mice^27^.

Earlier investigations in our laboratory have shown that intrathecal administration of ODN 2088 to mice sustaining a severe mid-thoracic contusion injury improves histopathological and functional outcomes^20,26^. These studies solely focused on changes occurring at the epicenter and analyzed the effects of the antagonist on the modulation of the infiltrating immune system cells, the expression of cytokines, white matter sparing and lesion volume^20,26^. However, treatments that target mechanisms at the epicenter could also alleviate pathology in remote regions, not only as a secondary consequence of effects at the lesion site, but through direct actions on cells at these distant regions. As the influence of the TLR9 antagonist at regions remote from the epicenter has not been adequately studied, the present investigation assessed the effects of ODN 2088 on the LDH.

## Results

### Intrathecal ODN 2088 treatment restores astroglial glutamate transporter levels in the LDH following SCI

We assessed the modulation of GLAST, GLT1 and EAAC1 levels in the LDH following SCI and after intrathecal treatment of injured mice with ODN 2088.

Glutamate transporter levels were analyzed at an acute (8 days post-injury [dpi]) and a subacute (28 dpi) time point to establish the progression of changes. In addition, our goal was to determine whether treatment with ODN 2088 during the acute phase is sufficient to observe the effects of the antagonist or an extended treatment into a subacute phase is necessary to reveal the outcomes of ODN 2088 administration.

**Figure 1:**
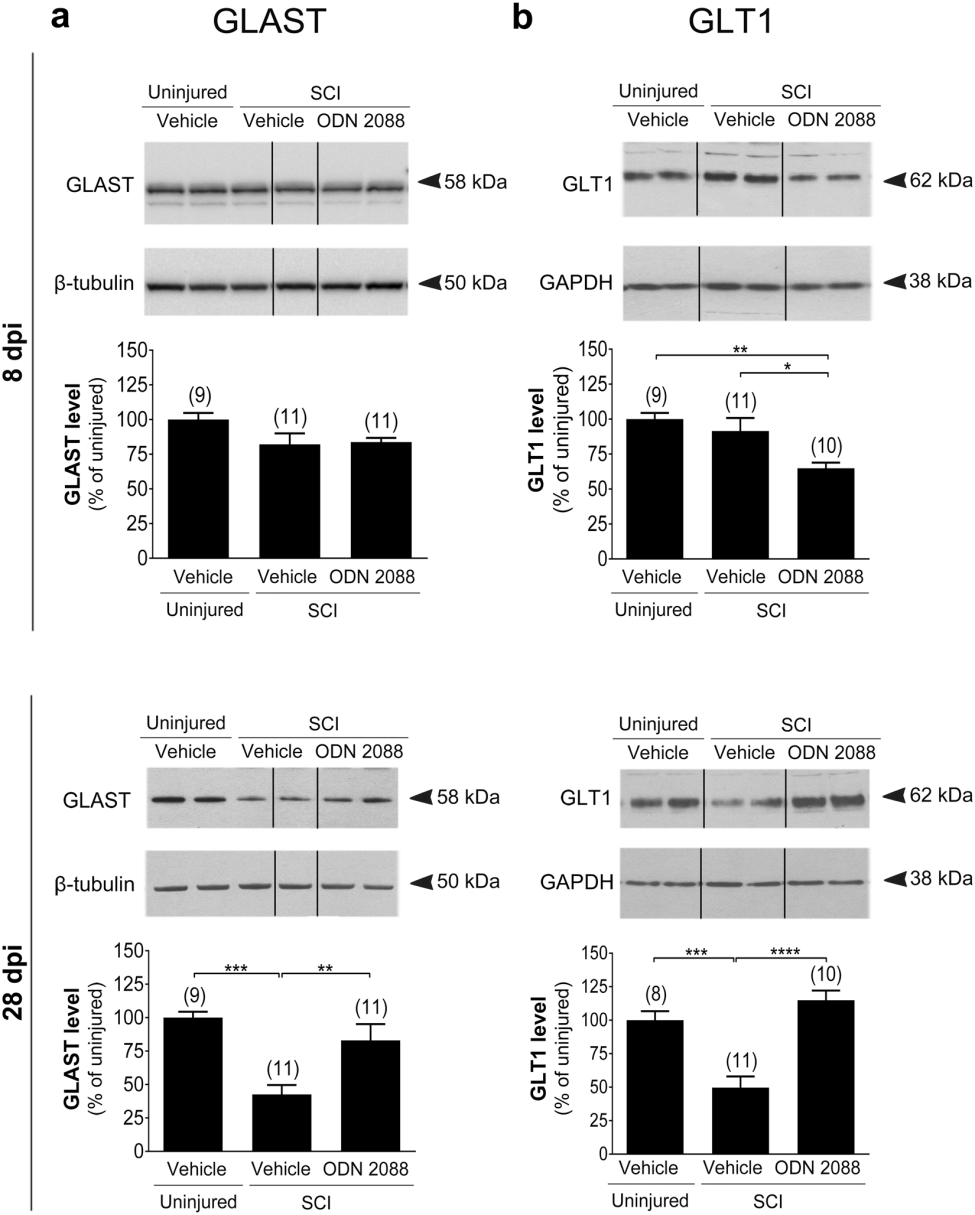
Effects of ODN 2088 on glial glutamate transporters in the LDH following thoracic SCI. (**a**) GLAST and (**b**) GLT1 protein levels. Upper panels show representative western blots (two representative lanes per treatment group) and lower panels are the graphic representation of the intensity of the bands after normalization to β-tubulin or GAPDH, which were used as a control for experimental variations. The dividing lines delineate the lanes that were cropped from each western blot. The same exposure was applied equally across the entire image. The original pictures of the full-length western blots can be found in Supplementary Fig. 10a,b. Values are mean ± S.E.M. The number of mice in each group is shown in parentheses above bars. Significantly different by one-way ANOVA with Tukey post-hoc test, *p < 0.05, **p < 0.01, ***p < 0.001, ****p < 0.0001.

At 8 dpi, GLAST levels in vehicle-treated uninjured mice were not statistically different than vehicle- or ODN 2088-treated injured mice (Fig. 1a). Similarly, SCI did not elicit any change in GLT1 protein levels compared to the uninjured mice (Fig. 1b) However, GLT1 levels were significantly reduced following administration of ODN 2088 to injured mice. To determine whether the ODN 2088-elicited reduction in GLT1 is observed only in injured mice or occurs even in the absence of injury, we treated uninjured mice with the antagonist over a period of 8 days. ODN 2088 significantly decreased GLT1 levels in the LDH of uninjured mice (Supplementary Fig. 1). These results lead to the conclusion that the ODN 2088–induced reduction in GLT1 levels at 8 dpi does not occur in the context of SCI.

At 28 dpi, GLAST levels were reduced by 57.4% in vehicle-treated injured mice as compared to vehicle-treated uninjured controls. ODN 2088 treatment restored GLAST levels to those of uninjured controls (Fig. 1a; p < 0.01). Similarly, vehicle-treated injured mice exhibited a 50.3% reduction in GLT1 levels as compared to those of vehicle-treated uninjured mice (Fig. 1b; p < 0.001). GLT1 levels were restored to uninjured values following treatment of injured mice with ODN 2088. To determine whether the ODN 2088-mediated upregulation of GLAST and GLT1 at 28 dpi is an injury-dependent effect or occurs even in the absence of injury, we treated uninjured mice with ODN 2088 or vehicle for 28 days and assessed glutamate transporter protein levels in the LDH. Following ODN 2088 treatment, GLAST protein levels within the intact LDH are reduced (Supplementary Fig. 2a) whereas GLT1 levels remain unaltered (Supplementary Fig. 2b). Taken together, the results indicate that ODN 2088 can regulate GLAST and GLT1 levels in both the uninjured and injured SC. However, in the uninjured SC, it downregulates GLAST and GLT1 levels at different times post-treatment, whereas in the injured SC, it upregulates GLAST and GLT1 levels and reverses the injury-elicited decrease in glutamate transporters. Therefore, the effects of ODN 2088 on glial glutamate transporters appear to be context-dependent.

In contrast to GLAST and GLT1, there was no difference in EAAC1 protein levels across treatment groups at either time point post-injury (Supplementary Fig. 3). Thus, in our experimental paradigm, both SCI and ODN 2088 treatment modulated glial, but not neuronal, glutamate transporters.

**Figure 2:**
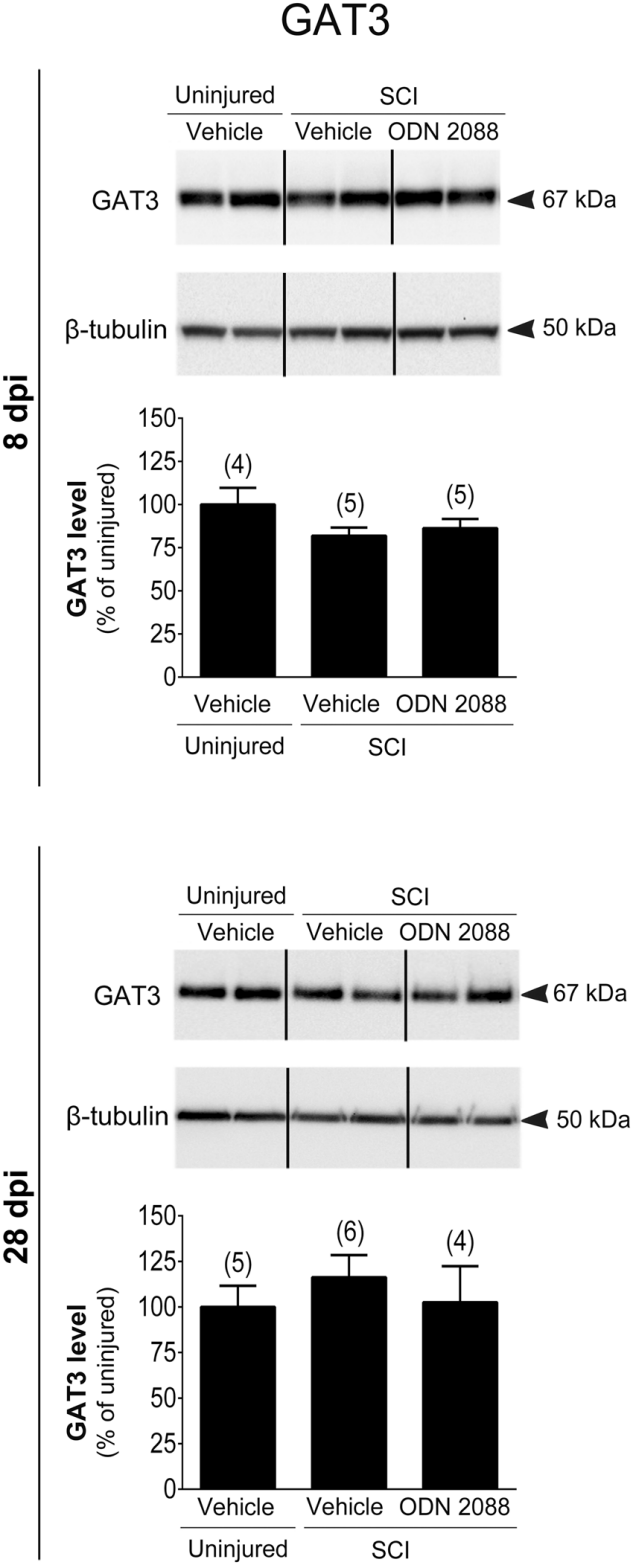
Effects of ODN 2088 on GAT3 expression at 8 dpi and 28 dpi in the LDH. Upper panels show representative western blots (two representative lanes per treatment group) and lower panels are the graphic representation of the intensity of the bands after normalization to β-tubulin, which was used as a control for experimental variations. The dividing lines delineate the lanes that were cropped from each western blot. The same exposure was applied equally across the entire image. The original pictures of the full-length western blots and gels can be found in Supplementary Fig. 10f. Values are mean ± S.E.M. The number of mice in each group is shown in parentheses above bars. No significant differences by one-way ANOVA.

To determine whether the effects of ODN 2088 are selective for GLAST and GLT1, we also analyzed the protein levels of gamma aminobutyric acid (GABA) transporter 3 (GAT3), which is primarily expressed by astrocytes^28^. GAT3 expression in the LDH was not altered by SCI or by ODN 2088 treatment of injured mice at either 8 or 28 dpi (Fig. 2), suggesting that the effects of ODN 2088 are selective for glial glutamate transporters.

### ODN 2088 treatment does not restore SCI-elicited alterations in glutamate receptor levels in the LDH

We undertook further studies to determine whether ODN 2088 has additional effects on the glutamatergic system in the LDH following SCI. To this end, we chose to analyze several glutamate receptors since they assume important roles in synaptic transmission in the DH^14^ and are expressed in both neurons^29–31^ and astrocytes^32,33^. We evaluated N-methyl-D-aspartate (NMDA) receptor subunits GluN2A and GluN2B, α-amino-3-hydroxy-5-methyl-4-isoxazolepropionic acid (AMPA) receptor subunits GluA1 and GluA2, and the metabotropic glutamate receptor mGluR1 in uninjured mice and injured mice treated with vehicle or ODN 2088.

**Figure 3:**
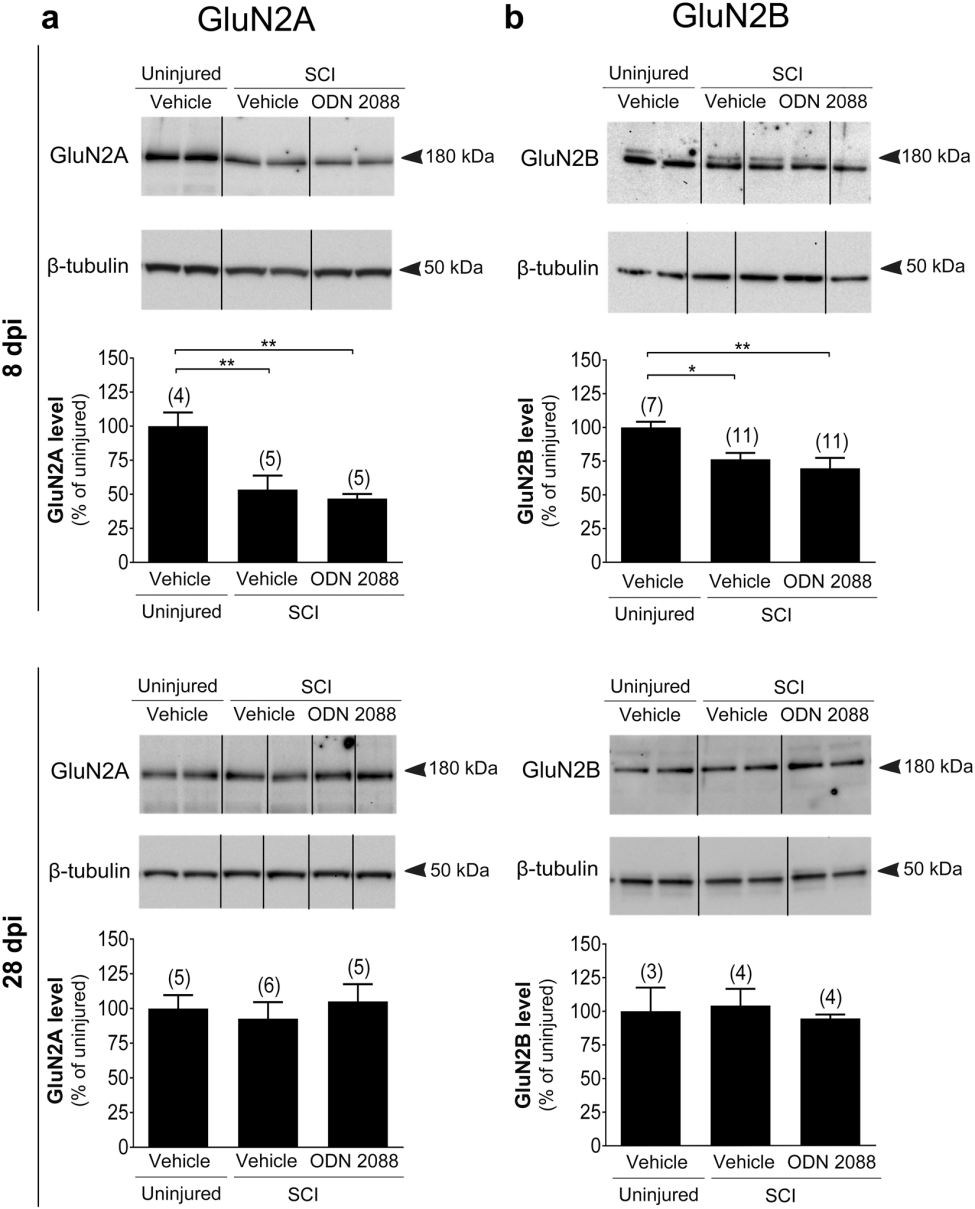
Effects of ODN 2088 on NMDAR expression in the LDH at 8 dpi and 28 dpi (**a**) GluN2A and (**b**) GluN2B protein levels. Upper panels show representative western blots (two representative lanes per treatment group) and lower panels are the graphic representation of the intensity of the bands after normalization to β-tubulin, which was used as a control for experimental variations. The dividing lines delineate the lanes that were cropped from each western blot. The same exposure was applied equally across the entire image. The original pictures of the full-length western blots can be found in Supplementary Fig. 10g,h. Values are mean ± S.E.M. The number of mice in each group is shown in parenthesis above bars. Significantly different by one-way ANOVA with Tukey post-hoc test, *p < 0.05, **p < 0.01.

**Figure 4:**
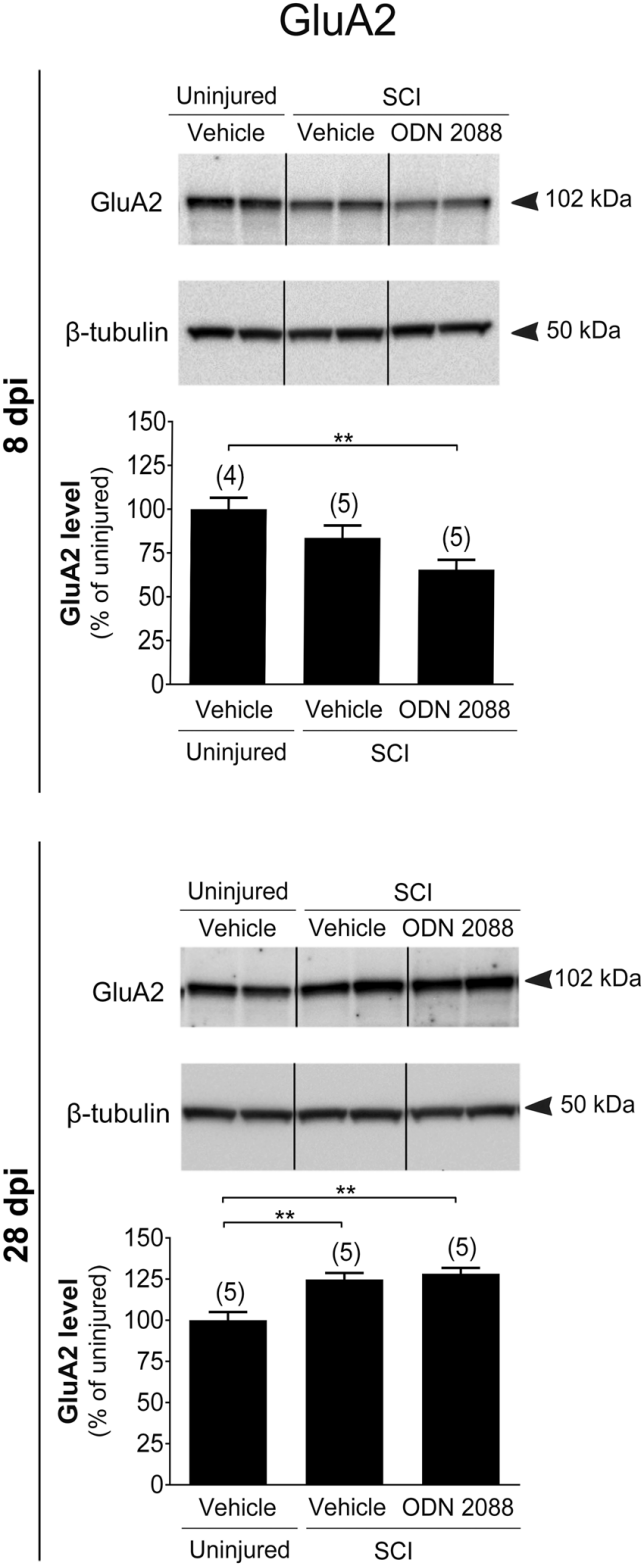
GluA2 expression in the LDH at 8 dpi and 28 dpi following ODN 2088 treatment. Upper panels show representative western blots (two representative lanes per treatment group) and lower panels are the graphic representation of the intensity of the bands after normalization to β-tubulin, which was used as a control for experimental variations. The dividing lines delineate the lanes that were cropped from each western blot. The same exposure was applied equally across the entire image. The original pictures of the full-length western blots can be found in Supplementary Fig. 10j. Values are mean ± S.E.M. The number of mice in each group is shown in parenthesis above bars. Significantly different by one-way ANOVA with Tukey post-hoc test, **p < 0.01.

**Figure 5:**
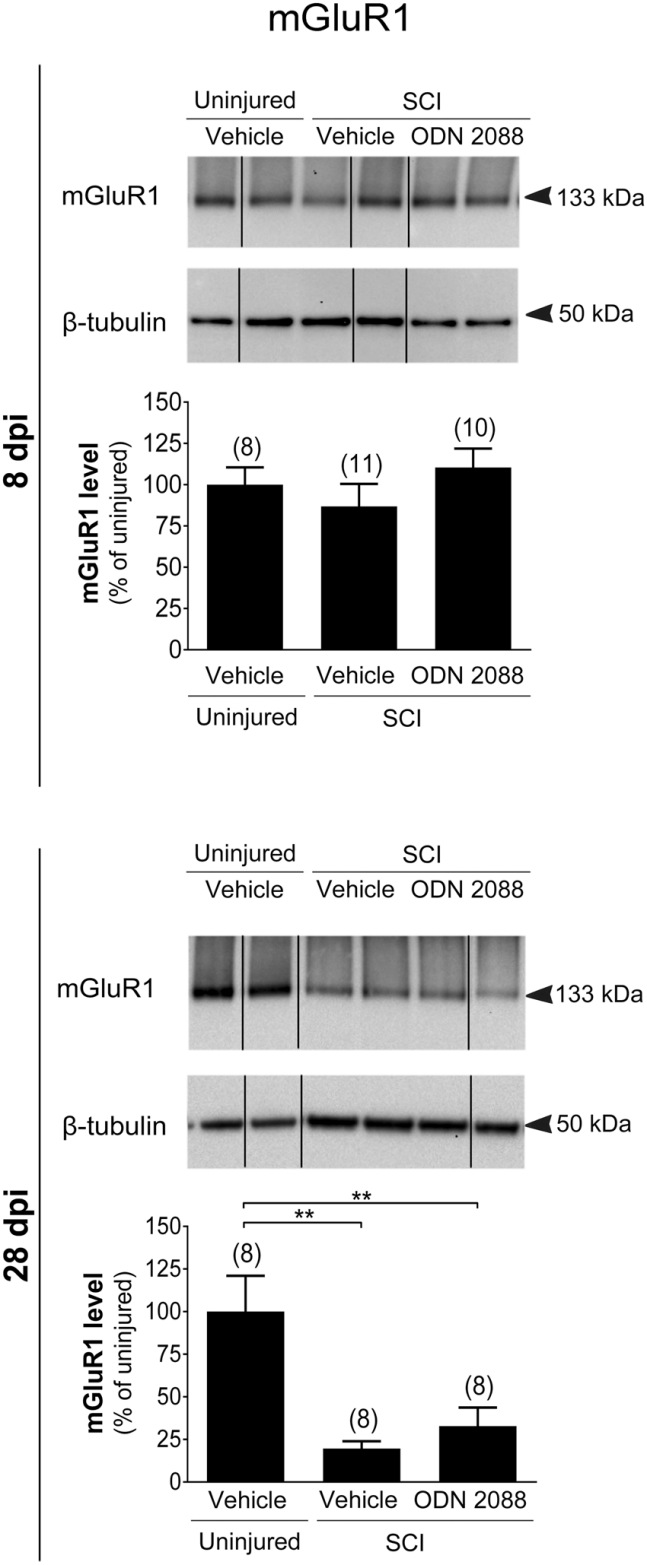
Effects of ODN 2088 on mGluR1 expression at 8 dpi and 28 dpi in the LDH. Upper panels show representative western blots (two representative lanes per treatment group) and lower panels are the graphic representation of the intensity of the bands after normalization to β-tubulin, which was used as a control for experimental variations. The dividing lines delineate the lanes that were cropped from each western blot. The same exposure was applied equally across the entire image. The original pictures of the full-length western blots can be found in Supplementary Fig. 10k. Values are mean ± S.E.M. The number of mice in each group is shown in parenthesis above bars. Significantly different by one-way ANOVA with Tukey post-hoc test, **p < 0.01.

At 8 dpi, GluN2A (Fig. 3a) and GluN2B (Fig. 3b) levels were transiently reduced in the LDH but returned to uninjured values by 28 dpi. ODN 2088 treatment did not have any effects at either time point. AMPA receptor subunit GluA1 was neither modulated by SCI nor ODN 2088 treatment at 8 or 28 dpi (Supplementary Fig. 4). AMPA receptor GluA2 levels in injured mice were similar to those of uninjured controls at 8 dpi but were significantly increased by SCI at 28 dpi (Fig. 4). However, ODN 2088 treatment had no further effects compared to vehicle-treated injured mice at either time point. Finally, we analyzed mGluR1 levels. There were no statistical differences in mGluR1 levels between the three groups at 8 dpi whereas SCI elicited a significant decrease in mGluR1 at 28 dpi although ODN 2088 did not restore these levels (Fig. 5).

The lack of ODN 2088 effects on the protein expression of several glutamate receptors further supports the notion that the modulation of GLAST and GLT1 levels by the antagonist is a selective effect on glial glutamate transporters in the LDH.

### Restoration of GLAST and GLT1 by ODN 2088 in the LDH does not coincide with alterations in astroglial activation

**Figure 6:**
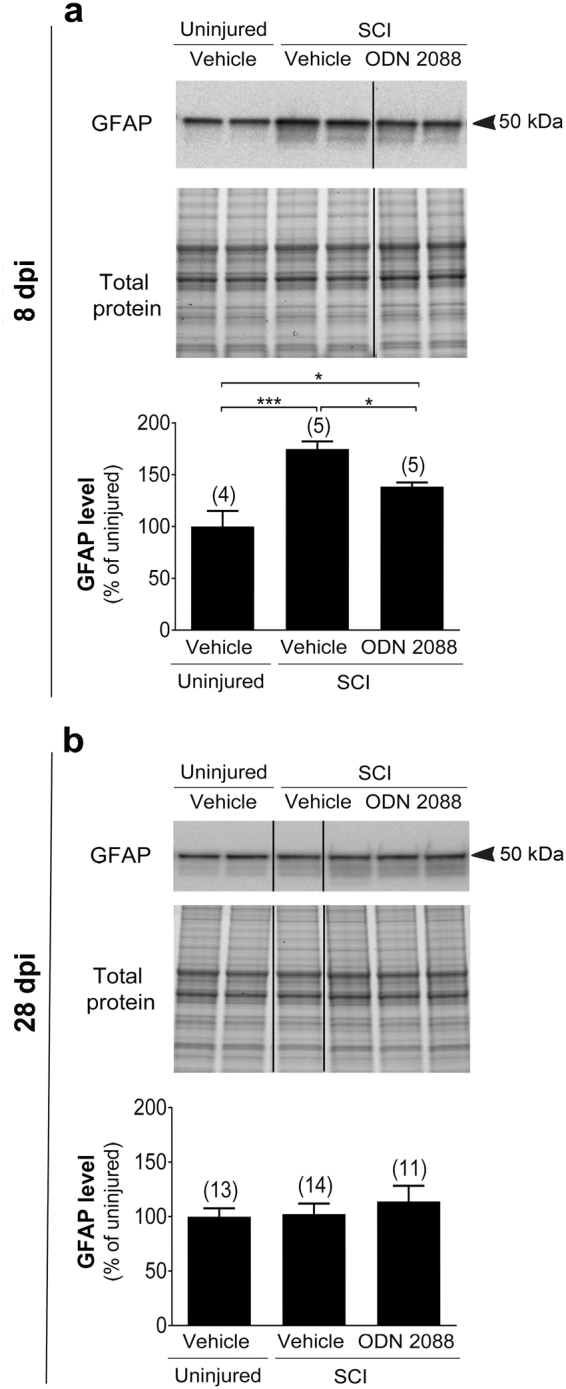
GFAP expression in the LDH of mice sustaining a mid-thoracic SCI and treated with ODN 2088. Protein levels at (**a**) 8 dpi and (**b**) 28 dpi. Upper panels show representative western blots (two representative lanes per treatment group) and lower panels are the graphic representation of the intensity of the bands after normalization to total protein. The dividing lines delineate the lanes that were cropped from each western blot. The same exposure was applied equally across the entire image. The original pictures of the full-length western blots and gels can be found in Supplementary Fig. 10l. Values are mean ± S.E.M. The number of mice in each group is shown in parentheses above bars. Significant differences by one-way ANOVA followed by Tukey post hoc, *p < 0.05, ***p < 0.001.

As astrocytes are the main cell type that express GLAST and GLT1^16,17^ and increased glial fibrillary acidic protein (GFAP) immunoreactivity has been reported in the LDH following thoracic SCI^4,5,34^ we assessed whether the restorative effects of ODN 2088 on GLAST and GLT1 are the outcome of a global change in astrocyte activation in response to the antagonist. We postulated that persistent astrocyte activation in the LDH of injured mice during the subacute phase could have led to the decrease in glutamate transporter expression and ODN 2088 could have restored GLAST and GLT1 levels through the attenuation of astrocyte activation. An increase in GFAP expression^35^ and morphological changes, characterized by cell body hypertrophy and process remodeling, have traditionally been used as markers of astrocyte activation^36^. Therefore, we evaluated GFAP levels in the LDH by western blotting and analyzed the morphology of GFAP immunoreactive cells. Western blots indicated that GFAP levels in the LDH of injured mice are increased compared to uninjured controls and ODN 2088 treatment significantly attenuates the increase in GFAP levels at 8 dpi (Fig. 6a). At this time, we did not observe any discernable cell body hypertrophy or alterations in the morphology of GFAP-positive processes in vehicle- or ODN 2088-treated mice compared to uninjured controls (Supplementary Fig. 5). At 28 dpi, the time when GLAST and GLT1 levels are restored by the antagonist, there were no significant differences in GFAP levels between vehicle-treated uninjured controls and vehicle- or ODN 2088-treated injured mice, as indicated by western blotting (Fig. 6b). The morphology of GFAP-positive cells remained similar across the three groups (Supplementary Fig. 5). These studies support the notion that injury- or ODN 2088-elicited changes in glutamate transporter expression do not coincide with increased GFAP expression or altered astrocyte morphology, hallmarks of cellular activation. Therefore, we propose that ODN 2088-elicited restoration of GLAST and GLT1 is not the consequence of a global effect on astrocyte activation.

**Figure 7:**
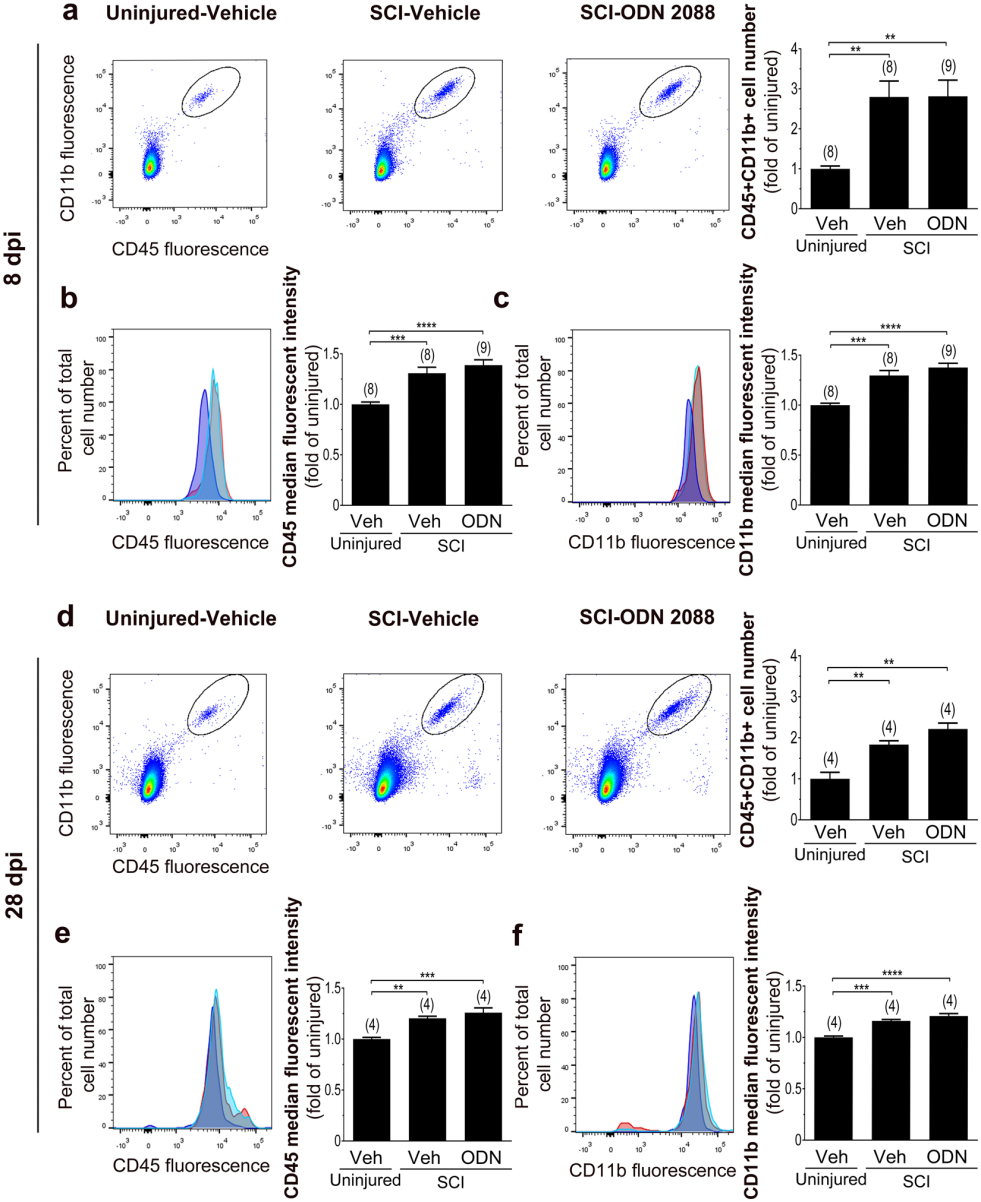
Effects of ODN 2088 on CD45^+^CD11b^+^ cells in the LDH following a mid-thoracic SCI at 8 dpi and 28 dpi. (**a**) The left panels are scatterplots showing CD45^+^CD11b^+^ cells while the right panel is the graphic representation of CD45^+^CD11b^+^ cell number (Uninjured-vehicle: 595.4 ± 126.3; SCI-vehicle: 1,447 ± 223.7; SCI-2088: 1,490 ± 198.3) and (**b**) Fluorescent intensity (FI) of CD45^+^ and (**c**) CD11b^+^ cells obtained from the LDH at 8 dpi. (**d**) The left panels are scatterplots showing CD45^+^CD11b^+^ cells while the right panel is the graphic representation of CD45^+^CD11b^+^ cell number (Uninjured-vehicle: 567.3 ± 89.4; SCI-vehicle: 1,040.5 ± 53.3; SCI-2088: 1,257.5 ± 80.8) and (**e**) Fluorescent intensity (FI) of CD45^+^ and (**f**) CD11b^+^ cells obtained from the LDH at 28 dpi. Dark blue histograms represent the FI of cells obtained from uninjured mice, the red histograms represent the FI of cells obtained from vehicle-treated injured mice, and the light blue histogram represent the FI of cells derived from ODN 2088-treated injured mice. Values are mean ± S.E.M. The number of mice in each group is indicated in parentheses above bars. Significantly different by one-way ANOVA, Tukey post-hoc test, **p < 0.01, ***p < 0.001, ****p < 0.0001.

Studies have also shown an increase in the levels of OX-42, a microglial marker, in the rat LDH following thoracic SCI^3,4,37^. Therefore, we investigated whether the restoration of GLAST and GLT1 coincides with an overall downregulation of microglial reactivity. We postulated that ODN 2088 could alter microglia number and activity in the LDH following SCI, and this, in turn, could change the LDH milieu leading to restoration of GLAST and GLT1 levels. We used flow cytometry to analyze the number and activation state of CD45^+^CD11b^+^ cells, presumably microglia/macrophages^38^, in the LDH. There was a significant increase in the number of CD45^+^CD11b^+^ cells in mice sustaining a SCI and treated with vehicle as compared to uninjured mice at both 8 and 28 dpi (by 2.8- and 1.8-fold, respectively) (Fig. 7a,d). ODN 2088 treatment did not affect the CD45^+^CD11b^+^ cell number. The median fluorescent intensity (MFI) of CD45 and CD11b, which is an index of cellular activation, was significantly increased following SCI, but not affected by ODN 2088 treatment at either time point (Fig. 7b,c,e,f). We also examined the effects of ODN 2088 on microglia morphology following SCI and ODN 2088 treatment by immunocytochemistry utilizing an antibody against ionized calcium binding adaptor molecule 1 (Iba-1), a microglia/macrophage marker. Under physiological conditions quiescent microglia exhibit a characteristic ramified morphology, with a small cell body and many thin and branched processes^39^. In contrast, activated microglia have thick and short processes or exhibit a round, macrophage-like morphology^40,41^. We did not observe any noticeable change in the ramified morphology of Iba-1 immunoreactive cells in the LDH of injured mice treated with vehicle or ODN 2088 at either 8 or 28 dpi (Supplementary Fig. 6) compared to uninjured controls. We also did not observe Iba-1 immunopositive cells with round, macrophage-like morphology, ruling out the possibility of infiltration of peripheral macrophages. To further assess the potential infiltration of other immune cells, which would have responded to ODN 2088 treatment, we quantified the number of GR-1+ and CD3+ cells, neutrophils and T-lymphocytes^42^, respectively, by flow cytometry. We did not find any significant neutrophil or T-lymphocyte infiltration in the LDH following SCI at 8 dpi (Supplementary Fig. 7) or 28 dpi (Supplementary Fig. 8). In contrast, in the injury epicenter, which was included in the studies as a positive control, GR-1+ and CD3+ cell number was significantly higher than those observed in uninjured controls. These results ascertained that infiltration of neutrophils or T-lymphocytes to the LDH is negligible and ruled out the possibility that the ODN 2088 effects are mediated through actions on these cells.

### Effects of ODN 2088 on growth factor and cytokine expression in the LDH

We next analyzed the expression of effectors reported to modulate GLAST and GLT1 expression. Since transforming growth factor-α (TGF-α)^43,44^, TGF-β1^45^, brain-derived neurotrophic factor (BDNF)^46^, and epidermal growth factor (EGF)^43,44,47^ are among factors that upregulate GLAST and/or GLT1 expression, we postulated that the restoration of GLAST and GLT1 levels at 28 dpi in the DH of ODN 2088-treated injured mice could be mediated by enhanced growth factor expression.

**Figure 8:**
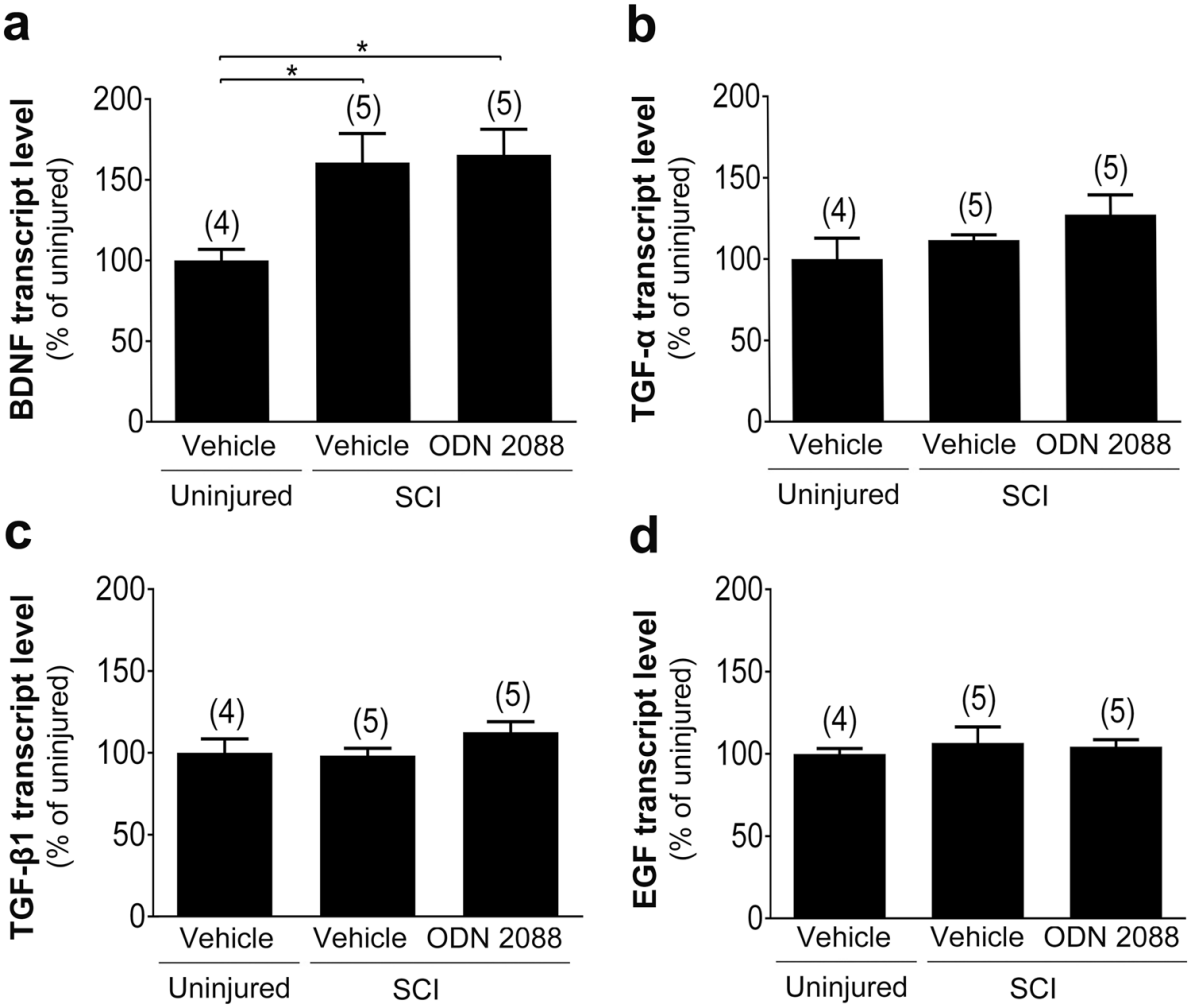
Effects of ODN 2088 on growth factor expression in the LDH at 28 dpi. (**a**) BDNF, (**b**) TGF-α, (**c**) TGF-β1, and (**d**) EGF transcript levels evaluated by qRT-PCR. Values represent mean ± S.E.M. The number of mice in each group is shown in parentheses above bars. Significantly different by one-way ANOVA, Tukey post-hoc test, *p < 0.05.

Quantitative RT-PCR indicated that BDNF transcript levels were significantly increased by 60.7% (p < 0.05) in vehicle-treated injured mice as compared to vehicle-treated uninjured controls (Fig. 8a). However, ODN 2088 treatment did not significantly alter BDNF transcript levels compared to those observed in vehicle-treated injured mice. Moreover, TGF-α, TGF-β1, and EGF transcript levels were comparable in vehicle-treated uninjured mice and vehicle- or ODN 2088-treated injured mice (Fig. 8b–d).

**Figure 9:**
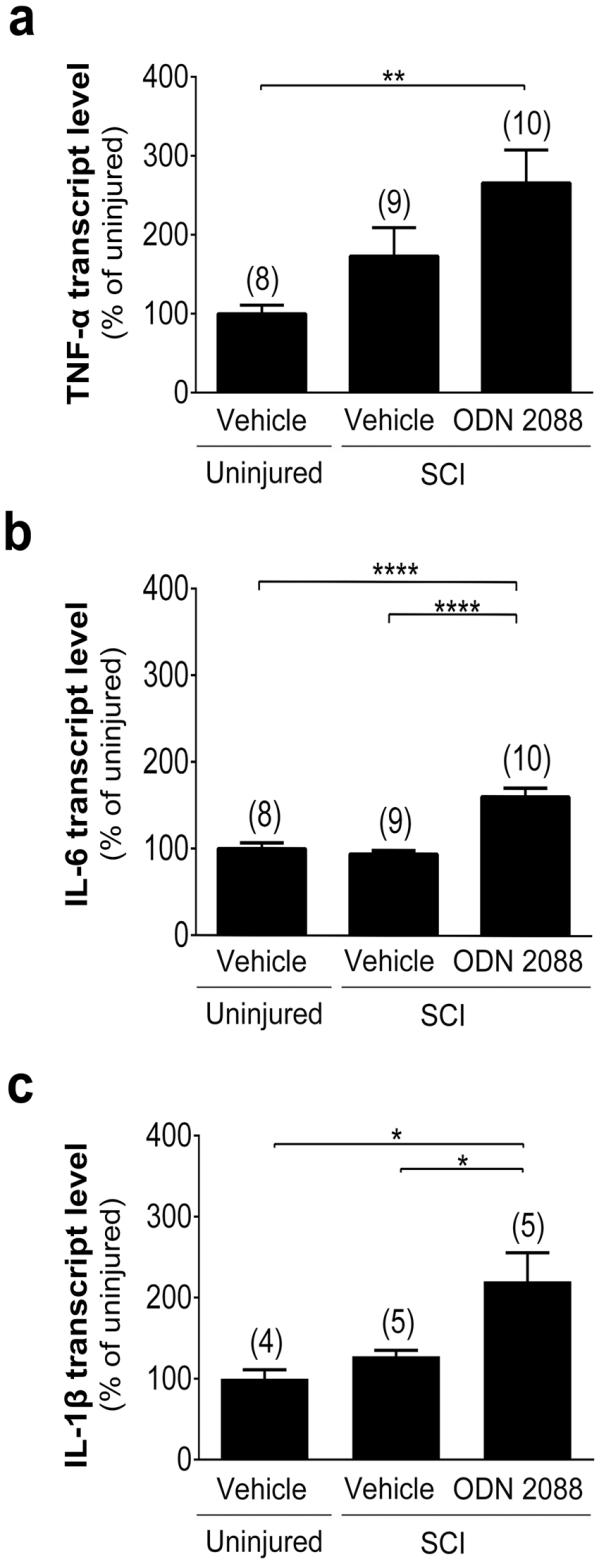
Effects of ODN 2088 on cytokine expression in the LDH at 28 dpi. (**a**) TNF-α, (**b**) IL-6, and (**c**) IL-1β transcript levels evaluated by qRT-PCR. Values represent mean ± S.E.M. The number of mice in each group is shown in parentheses. Significantly different by one-way ANOVA, Tukey post-hoc test, *p < 0.05, **p < 0.01, ****p < 0.0001.

Subsequently, we considered an alternative mechanism that focused on cytokines. Because tumor necrosis factor-α (TNF-α)^48–51^, interleukin-6 (IL-6)^52^, and IL-1β^53^ downregulate GLAST and/or GLT1 expression, we postulated that an increase in these cytokines in the DH of injured mice could have caused the decrease in GLAST and GLT1 at 28 dpi and ODN 2088 treatment could have restored the levels of the glutamate transporters by reducing the expression of these cytokines. To assess this hypothesis, we quantified TNF-α, IL-6, and IL-1β transcript levels in the DH of vehicle-treated uninjured and injured mice as well as ODN 2088-treated injured mice. TNF-α transcript levels were comparable in vehicle- and ODN 2088-treated injured mice (Fig. 9a). Surprisingly and contrary to our expectation, ODN 2088 treatment induced a significant increase in both IL-6 (60.5%; p < 0.0001) and IL-1β (120.2%; p < 0.05) transcript levels compared to vehicle-treated injured or uninjured mice (Fig. 9b,c).

## Discussion

The present studies show that a mid-thoracic contusion injury reduces glial glutamate transporter GLAST and GLT1 protein levels within the LDH at 28 dpi and intrathecal treatment of injured mice with a TLR9 antagonist restores GLAST and GLT1 levels to uninjured values. Moreover, our findings suggest that the antagonist selectively affects astroglial glutamate transporters, as the expression of the neuronal glutamate transporter EAAC1 and the astroglial GABA transporter GAT 3 were not altered by the administration of ODN 2088 to injured mice. The restoration of GLAST and GLT1 does not appear to be a global effect of ODN 2088 on astrocyte activation or the microglial reaction in the LDH. Thus, we propose that in the LDH of mice sustaining a mid-thoracic injury, the effects of ODN 2088 are restricted to select mechanisms with astroglial glutamate transporters being one of the targets. This idea is further supported by the lack of ODN 2088 effects on glutamate receptors in the DH following SCI.

Investigations by other laboratories also indicate that GLAST and GLT1 levels in the DH are downregulated following injury. A decrease in GLAST and GLT1 in caudal regions immediately adjacent to the epicenter has been reported^54–59^. In particular, Olsen *et al*.^56^ showed that the reduction in GLT1 persists until 4 weeks post-injury in the rat. The downregulation of GLAST and GLT1 levels in the DH has been implicated in the development of neuronal hyperexcitabililty following SCI. Subsequent to a cervical contusion, the overexpression of GLT1 in astrocytes, located in the DH caudal to the injury site, attenuated the over-activation of DH neurons in mice^60^. Even though our investigations focused on a caudal region considerably remote from the injury epicenter, our findings are consistent with the aforementioned reports showing a decrease in GLAST and GLT1 in the DH following SCI. However, our results and those reported by Kim *et al*.^54^ differ, as these authors did not observe a decrease in GLAST and GLT1 levels in lumbar segments L1-L2 and L4-L5 following a T12 contusion, at 4 weeks post-injury. These seemingly disparate findings may stem from the fact that Kim and colleagues^54^ analyzed glutamate transporters in the entire spinal segment containing both grey and white matter, whereas our studies quantified GLAST and GLT1 in the isolated DH and therefore, focused only on the grey matter. Species differences (rat versus mouse) as well as injury severity could also account for the dissimilar results.

How does ODN 2088 treatment restore the SCI-elicited decrease in GLAST and GLT1 levels? To address this issue, we considered a number of possibilities. First, we investigated whether ODN 2088 alters the cellular environment in the LDH. In our earlier report, we showed that ODN 2088 modifies the milieu at the lesion site by downregulating the number of inflammatory cells including local microglia/macrophages^20^. However, these earlier studies had not analyzed how the TLR9 antagonist affects regions remote from the epicenter, even though it was administered by intrathecal injection and could have affected the lumbar region. Although the number and activation of microglia/macrophages in the LDH was increased at 28 dpi, neither of these parameters was reduced following treatment with the antagonist. Therefore, we propose that the modulation of GLAST and GLT1 by ODN 2088 is not due to a change in the microglial reaction in the LDH.

In the current study, GFAP level, an indicator of astrocyte activation, was upregulated in the LDH at 8 dpi, even though the morphology of the GFAP immunoreactive cells appeared to be similar to those of uninjured controls. Yet, GLAST and GLT1 levels in vehicle-treated injured mice remained comparable to those of uninjured mice, suggesting a disconnect between astrocyte activation and glial glutamate expression. Moreover, at 8 dpi, the reduction in GLT1 levels in response to ODN 2088 treatment did not occur in the context of SCI, as it was also observed in uninjured mice treated with the antagonist for 8 days. At 28 dpi, GFAP levels and astrocyte morphology were comparable in all the three experimental groups, suggesting that astrocytes were not in an activated state. Interestingly, the strongest modulation of GLAST and GLT1 by SCI and ODN 2088 treatment occurred at 28 dpi, at which time GLAST and GLT1 were reduced in vehicle-treated injured mice and restored to uninjured values in ODN 2088-treated injured mice. The upregulation of GLAST and GLT1 following ODN 2088 treatment occurred in the context of SCI since administration of the antagonist to uninjured mice did not increase GLAST or GLT1 expression. These results, taken together, suggest a dissociation between astrocyte activation and GLAST and GLT1 protein expression. Our findings are in agreement with investigations from other laboratories showing a dissociation between astrocyte activation and glutamate transporter expression^53,59^.

It is worth noting that the upregulation of GFAP in the LDH at an acute injury phase is in agreement with studies in the rat showing increased GFAP immunoreactivity^5^. However, in sub-acute SCI, our results concur with those of Detloff *et al*.^3^ who found no evidence of astrocyte hypertrophy or increased GFAP in the L5 DH of the rat at 35 days following thoracic injury, whereas they do not agree with those of Gwak and colleagues^5^ who reported increased GFAP immunoreactivity in the LDH of the rat at 30 days following thoracic injury.

Subsequently, we assessed whether there were alterations in the expression of effectors reported to modulate GLAST and GLT1 expression. Initially, we proposed that the restoration of GLAST and GLT1 following treatment of injured mice with ODN 2088 could be the result of increased growth/neurotrophic factor expression since BDNF, EGF, TGF-β1 and TGF-α upregulate GLAST and/or GLT1 levels^43–47^. However, our results did not support this hypothesis. Subsequently, we postulated that an increase in cytokine expression in the LDH of injured mice downregulates GLAST and GLT1 levels and, ODN 2088, similar to its effects at the epicenter^20^, suppresses the injury-elicited increase in cytokine expression, leading to the restoration of GLAST and GLT1 levels. We focused on TNF-α, IL-6 and IL-1β because studies have shown that these cytokines downregulate GLAST and/or GLT1 expression. TNF-α reduces GLAST levels in rat astrocytes^49,50^ and human fetal astrocytes, *in vitro*^51^. A link between IL-6 and GLT1 has also been described, as IL-6 blocking antibodies reversed the decrease in GLT1 levels observed in SCI-induced pain^52^. Moreover, in a model of encephalomyelitis, increased IL-1β levels paralleled a reduction in spinal GLT1 protein levels and IL-1β inhibitors prevented the decrease in GLT1^53^. However, contrary to our postulate, we did not find an increase in TNF-α, IL-1β, IL-6 transcript levels in the LDH at 28 dpi. Our results with TNF-α and IL-1β are in agreement with an earlier report showing that protein levels of these cytokines in the dorsal lumbar SC are comparable to baseline values at 35 dpi in rats sustaining a thoracic contusion injury, even though their levels are transiently increased up to 3 weeks post-injury^3^. However, our result with IL-6 contrasted the report by Detloff and colleagues^3^, which showed a sustained increase in IL-6 even at 35 dpi. Multiple factors such as species differences (rat versus mouse) and quantification of protein versus transcript levels could have contributed to this discrepancy. Surprisingly, treatment of injured mice with ODN 2088 resulted in an increase, rather than a decrease, in IL-1β and IL-6 transcript levels. Thus, our results show a dissociation between GLAST and GLT1 levels in the LDH and IL-1β and IL-6 expression. The mechanism underlying the unexpected upregulation of IL-1β and IL-6 transcript levels in response to ODN 2088 is not yet clear. The increase in these transcript levels may result from the overall effect of ODN 2088 on multiple cell types, including neurons, which also produce IL-1β^61^ and IL-6^62^ and have been demonstrated to respond to ODN 2088 *in vitro*^18^.

The aforementioned results ruled out a number of mechanisms as possible mediators of ODN 2088-elicited restoration of GLAST and GLT1 in the LDH following SCI. It is conceivable that the effects of the antagonist at regions remote from the epicenter could be secondary to the effects at the epicenter, since ODN 2088 attenuates the inflammatory reaction and increases white matter sparing at the lesion site^20,26^. Improved protection of cells and tissue at the epicenter could, in turn, limit the pathological changes in regions remote from the primary injury site. However, independent of the underlying mechanism, the present study shows that astroglial glutamate transporters are selective targets of ODN 2088 treatment in regions at a distance from the injury epicenter following SC trauma. Our findings highlight the potential of ODN 2088 to modulate mechanisms implicated in synaptic transmission^63^ below the injury level.

## Methods

### Animals

Eight to ten-week-old female C57BL/6 mice (Charles River Laboratories, Wilmington, MA) were housed in a pathogen-free barrier facility and maintained on a 12 hr dark/light cycle with food and water given *ad libitum*. Sentinels were housed in the same room and periodically checked for infection. All animal protocols and experiments were approved by the Institutional Animal Care and Use Committee at Rutgers University and were performed in accordance with relevant guidelines and regulations.

### Spinal cord injury (SCI)

Mice were anesthetized with Ketamine (80 mg/kg; Vedco, St. Joseph, MO) and Xylazine (10 mg/kg; Akorn Inc., Decatur, IL) that was administered via intraperitoneal (ip) injection. Following a laminectomy at thoracic level 8 (T8), a severe contusion injury (70 kilodyne force) was induced using the Infinite Horizon Impactor (Precision Systems and Instrumentation, Lexington, KY). Uninjured mice were anesthetized, received superficial skin incisions, and received wound clips to ensure blinded evaluations. Since a laminectomy does not induce major inflammatory changes in the SC (Supplementary Fig. 9), a surgical control was not included in the current studies. Immediately postoperatively, all mice received subcutaneous injections of Lactated Ringers solution (1 ml; Baxter, Deerfield, IL), Baytril (0.02 ml; Bayer, Kansas City, KS), and Buprenorphine (0.05 ml; Hospira, Lake Forest, IL). Antibiotics, analgesics, and 1 ml of normal saline (Baxter) were administered to mice twice daily for 7 days thereafter. Bladders were manually expressed twice per day. On day 1 post-injury (pi), hind limb locomotor function was assessed with the Basso Mouse Scale (BMS)^64^ to confirm that all mice received an injury of equivalent severity (BMS score ≤ 2), and that each uninjured mouse exhibited normal baseline stepping and coordination (BMS score = 9).

### Intrathecal delivery of ODN 2088 by lumbar puncture (LP)

Injured mice were randomized to receive either vehicle (endotoxin-free water) or ODN 2088 (150 ng/g body weight) via lumbar puncture (LP) based on the BMS scores obtained on day 1 pi. All injured mice had a BMS score ≤ 2 on 1 dpi and randomization was performed in a manner that the average injury severity in the vehicle- and ODN 2088-receiving groups was equivalent prior to initiation of the treatment. After mice were anesthetized with isoflurane (1.0 l/min at a concentration of 3.0% in oxygen), a 27-gauge needle attached to a Hamilton syringe was inserted into the subarachnoid space between lumbar vertebrae L5 and L6, and vehicle or ODN 2088 was delivered into the intrathecal space. The first treatment was given at 24 hrs pi and additional doses were administered every 48 hrs thereafter for the duration of the study. All uninjured mice received vehicle via an intrathecal injection.

### Open-field locomotor function

Hind limb locomotor function was assessed with the BMS^64^ on days 1, 7, 14, 21, and 28 pi by two observers blinded to the experimental conditions.

### Flow cytometry

On 8 dpi and 28 dpi, mice were perfused with saline. The SC was removed and the LDH was dissected out. In addition, a 3 mm segment containing the injury epicenter was collected. The tissue was minced in Hank’s Balanced Salt Solution (HBSS) (Thermo Fisher Scientific Inc., Rockford, IL) followed by treatment with trypsin (0.5 mg/ml; Thermo Fisher Scientific Inc.) and collagenase (1 mg/ml; Sigma, St. Louis, MO) in Dulbecco’s Modified Eagle Medium (DMEM) (Thermo Fisher Scientific Inc.). After being passed through a 40 μm strainer, cells were centrifuged and re-suspended in ACK lysing buffer (Quality Biological Inc., Gaithersburg, MD) for 3 min. Cells were then washed with Dulbecco’s Phosphate-Buffered Saline (DPBS; Corning, Corning, NY) followed by fluorescence-activated cell sorting (FACS) buffer (DPBS with 2% Fetal Bovine Serum (FBS) and 0.09% Sodium Azide). Samples were incubated with Fc Block for 10 min (1:25; BD Bioscience, Franklin Lakes, NJ) followed by 30 min with antibodies against GR-1, CD11b, CD45, and CD3e (Table 1) or isotype controls in FACS buffer. Finally, cells were washed with FACS Buffer and fixed with 4% paraformaldehyde in phosphate buffered saline (PF/PBS). Sample acquisition was performed using the BD LSRII Flow Cytometer and 750,000 events were collected per sample. The data were analyzed using FlowJo software (V 10.0.8; Tree Star Inc., Ashland, OR).

**Table 1:** Antibody probing conditions used for flow cytometry analyses.

Target	Form	Company	Host	Clone	Isotype	Dilution
CD45	APC	BD Bioscience (Franklin Lakes, NJ)	Rat	30-F11	Rat IgG2b, κ	1:100
CD11b	Alexa Fluor 488	BD Bioscience (Franklin Lakes, NJ)	Rat	M1/70	Rat IgG2b, κ	1:100
CD3e	PerCP-Cy5.5	BioLegend (San Diego, CA)	Hamster	145-2C11	Hamster IgG1, κ	1:100
GR-1 (Ly-6G/Ly-6C)	APC-Cy7	BioLegend (San Diego, CA)	Rat	RB6-8C5	Rat IgG2b, κ	1:100

### Immunohistochemistry (IHC)

On 8 dpi and 28 dpi, mice were euthanized by transcardial perfusion with saline followed by 4% paraformaldehyde in 0.1 M phosphate buffer (PF/PBS; pH = 7.4). Spinal cords were removed and post-fixed in PF overnight. They were cryoprotected in 27% sucrose/PBS, embedded in optimal cutting temperature (OCT), frozen in a dry ice-ethanol slurry, and sectioned on a cryostat. Transverse sections (30 µm) were obtained from equivalent regions of the lumbar SC, across all three treatment groups, thaw-mounted side-by-side on slides and processed concomitantly. The sections were blocked with 30% normal goat serum (NGS) in 10 mM PBS (pH = 7.4), containing 0.1% Triton X-100 for 1 hr at room temperature and incubated overnight in rabbit polyclonal antibodies against GFAP (1:1000; Agilent Technologies, Santa Clara, CA) or Iba1 (1:500; Wako Laboratory Chemicals, Richmond, CA). The sections were then rinsed in PBS and incubated with a goat anti-rabbit Alexa Fluor 488 secondary antibody (1:500; Thermo Fisher Scientific Inc.) for 1 hr at room temperature. The sections were rinsed again in PBS and cover-slipped with ProLong Diamond antifade mountant with DAPI (Thermo Fisher Scientific Inc.). Fluorescent images were captured on a Nikon A1R confocal microscope using NIS Elements AR 4.00.07 software. Images were assembled at 10× and 60× magnification.

### Protein extraction and western blot analysis

Mice were sacrificed by CO_2_ inhalation at 8 dpi and 28 dpi. Following decapitation, the SCs were removed and the LDH was dissected out. The tissue was fresh frozen and conserved at −80 °C. To extract protein, tissue was homogenized with a motorized pestle in 150–200 μl lysis buffer (pH = 8.0; 10 mM HEPES, 150 mM NaCl, 0.02% sodium azide, 0.1% sodium doedecyl sulfate, 0.5% deoxycholic acid, 50 mM NaF, and 1% NP40) to which a protease inhibitor cocktail was freshly added (1:100; P8340; Sigma). Samples underwent three freeze-thaw cycles followed by five rounds of vortexing for 15 sec each. Lysates were sonicated in a low-frequency ultrasonic bath for 15 sec and then centrifuged at 8,000 rpm for 1 min. The supernatant, containing cytosolic and membrane proteins, was collected and protein was quantified by the BCA assay (Thermo Fisher Scientific Inc.) according to the manufacturer’s instructions.

Proteins (20 µg/lane) were resolved on a 4–12% Criterion XT Bis-Tris gel or a stain-free 4–15% Criterion TGX gel (Bio-Rad) followed by electrophoresis at 200 V for 1 hr. Samples were electrotransferred onto a polyvinylidene difluoride (PVDF) membrane for 7 or 17 min at 25 V using the Trans-Blot Turbo transfer system (Bio-Rad) or for 1 hr at 100 V or 16 hrs at 10 V using the Criterion Blotter Cell (Bio-Rad). Tris buffered saline (20 mM Trizma Base, 500 mM NaCl, pH = 7.5) containing 0.05–0.1% Tween-20 (T-TBS) was used to prepare blocking buffer. Table 2 lists the blocking and probing conditions for each antibody used.

**Table 2:** Antibody probing conditions used for western blot analyses.

Target	Abbreviation	Company	Host	Blocking Conditions	Primary Antibody Conditions
Beta III Tubulin	β-tubulin III	Abcam (Cambridge, MA)	Rabbit	30 min in 5% milk in 0.05% T-TBS	1:30,000 in 5% milk in 0.05% T-TBS
Excitatory Amino Acid Carrier 1	EAAC1 (EAAT3)	AbBiotec (San Diego, CA)	Rabbit	1 hr in 5% milk in 0.05% T-TBS	1:20,000 in 5% milk in 0.05% T-TBS
GABA Transporter 3	GAT3	Novus Biologicals (Littleton, CO)	Rabbit	1 hr in 5% milk in 0.1% T-TBS	1:4000 in 5% milk in 0.1% T-TBS
Glial Fibrillary Acidic Protein	GFAP	Dako (Carpinteria, CA)	Rabbit	30 min in 5% milk in 0.05% T-TBS	1:30,000 in 5% milk in 0.05% T-TBS
Glutamate-Aspartate Transporter	GLAST (EAAT1)	Cell Signaling (Danvers, MA)	Rabbit	1 hr in 5% milk in 0.1% T-TBS	1:10,000 in 5% BSA in 0.1% T-TBS
Glutamate Receptor 1	GluA1 (GluR1)	Millipore (Billerica, MA)	Rabbit	30 min in 5% milk in 0.05% T-TBS	1:1000 in 5% milk in 0.05% T-TBS
Glutamate Receptor 2	GluA2 (GluR2)	Millipore (Billerica, MA)	Mouse	1 hr in 5% BSA in 0.1% T-TBS	1:1000 in 5% BSA in 0.1% T-TBS
Glutamate Transporter-1	GLT1 (EAAT2)	Novus Biologicals (Littleton, CO)	Rabbit	1 hr in 5% milk in 0.1% T-TBS	1:10,000 in 5% BSA in 0.1% T-TBS
Glyceraldehyde 3-phosphate dehydrogenase	GAPDH	Calbiochem (Billerica, MA)	Mouse	30 min in 5% milk in 0.05% T-TBS	1:30,000 in 5% milk in 0.05% T-TBS
Metabotropic Glutamate Receptor 1	mGluR1	BD Bioscience (Franklin Lakes, NJ	Mouse	30 min in 5% milk in 0.05% T-TBS	1:2500 in 5% milk in 0.05% T-TBS
N-methyl D-aspartate receptor subtype 2A	GluN2A (NR2A)	Millipore (Billerica, MA)	Rabbit	1 hr in 5% milk in 0.1% T-TBS	1:10,000 in 5% milk in 0.1% T-TBS
N-methyl D-aspartate receptor subtype 2B	GluN2B (NR2B)	Calbiochem (Billerica, MA)	Rabbit	30 min in 5% milk in 0.05% T-TBS	1:1000 in 5% milk in 0.05% T-TBS

To compensate for loading differences, membranes were stripped with Re-Blot Plus Western Blot Mild Antibody Stripping Solution (EMD Millipore Corporation, Billerica, MA) and re-probed with antibodies against the housekeeping proteins glyceraldehyde 3-phosphate dehydrogenase (GAPDH) or β-III-tubulin. Bands were visualized using Clarity Western ECL Blotting Substrate (Bio-Rad) or the Pierce ECL Western Blotting Substrate (Thermo Fisher Scientific Inc.) on film (Denville Scientific Inc., Holliston, MA) or the ChemiDoc Touch Imaging System (Bio-Rad) according to the manufacturer’s instructions. The intensity of the bands in each lane was measured using the UN-SCAN-IT gel Analysis Software (Silk Scientific, Orem, UT) and normalized to the intensity of the band for the housekeeping protein in the same lane. For lysates that were resolved on stain-free gels, the signal was normalized to total protein in each lane. Total protein was visualized using the ChemiDoc Touch Imaging System (Bio-Rad) and quantified using the UN-SCAN-IT software. This latter approach was used in some of the experiments because the housekeeping genes also appeared modulated.

### Quantitative reverse transcriptase-polymerase chain reaction (qRT-PCR)

On 28 dpi, mice were sacrificed by CO_2_ inhalation. Following decapitation, the SCs were removed and the LDH was dissected out. Tissue was frozen and conserved at −80 °C. Total RNA was isolated as described previously^20^ and treated with DNase I to remove contaminating genomic DNA (Thermo Fisher Scientific Inc.). 100 ng total RNA was reverse transcribed into cDNA using random hexamers (Thermo Fisher Scientific Inc.) and Superscript III reverse transcriptase (Life Technologies) in the presence of RNase inhibitor (Thermo Fisher Scientific Inc.). Real time-PCR was performed with a SYBR green master mix containing Rox (Clontech, Mountain View, CA) using Applied Biosystems 7500 Real-Time PCR Systems. Samples were heated at 95 °C for 10 min and amplified for 40 cycles with denaturation at 95 °C for 15 sec, annealing at 58 °C for 45 sec (60 °C for 30 sec for TNF-α), and extension at 72 °C for 30 sec (70 °C for 1 min for TNF-α). Samples were then heated at 95 °C for 15 sec and subsequently underwent a melting curve analysis from 60 °C to 95 °C. The threshold cycle number (C_T_) of each target was calculated and expressed relative to that of GAPDH. ΔΔC_T_ values of targets were then calculated and presented as relative fold induction. Primer sequences are provided in Table 3. All primers were used at a 0.1 µM concentration, with the exception of IL-6 (0.2 µM), and were purchased from Real Time Primers (Elkins Park, PA).

**Table 3:** Sequence of the primers used in qRT-PCR.

Growth factor or cytokine	Abbreviation	Forward Sequence	Reverse Sequence
Brain-Derived Neurotrophic Factor	BDNF	5′-GGT GCA GAA AAG CAA CAA GT-3′	5′-GCA CAA AAA GTT CCC AGA GA-3′
Epidermal Growth Factor	EGF	5′-TTG TTA GCA CCA TCC CTC AT-3′	5′-CGG GAG AGT TCT TTG TCT CA-3′
Glyceraldehyde 3-phosphate dehydrogenase	GAPDH	5′-CTG GAG AAA CCT GCC AAG TA-3′	5′-TGT TGC TGT AGC CGT ATT CA-3′
Interleukin-1β	IL-1β	5′-CCC AAC TGG TAC ATC AGC AC-3′	5′-TCT GCT CAT TCA CGA AAA GG-3′
Interleukin-6	IL-6	5′-CTA CCC CAA TTT CCA ATG CT -3′	5′-ACC ACA GTG AGG AAT GTC CA-3′
Transforming Growth Factor-α	TGF-α	5′-CAC TGG ACT TCA GCC CTC TA-3′	5′-TCC AGC AGA CCA GAA AAG AC-3′
Transforming Growth Factor-β1	TGF-β1	5′-GCT ACC ATG CCA ACT TCT GT-3′	5′-CGT AGT AGA CGA TGG GCA GT-3′
Tumor Necrosis Factor-α	TNF- α	5′- TTG GAG TCA TTG CTC TGT GA -3′	5′-GTC CCA GCA TCT TGT GTT TC-3′

### Statistical analysis

SPSS 20.1 and Graphpad statistical packages were used for all analyses. Two-tailed, independent t-tests or One-way ANOVAs followed by a Tukey’s post-hoc analysis were used. Data are presented as mean ± standard error of the mean (S.E.M).

### Data Availability

The data sets generated and/or analyzed during the current study are available from the corresponding author on reasonable request.

## Electronic supplementary material

Supplementary Information
