# Supplementary material for: A toll-like receptor 9 antagonist restores below-level glial glutamate transporter expression in the dorsal horn following spinal cord injury
Source: Sci Rep. 2018 Jun 7;8:8723. doi: 10.1038/s41598-018-26915-2 (PMC5992189; doi:10.1038/s41598-018-26915-2)
Supplement: Supplementary file 1 — Supplementary Information [file 41598_2018_26915_MOESM1_ESM.pdf]

Supplementary Information

A toll-like receptor 9 antagonist restores below-level glial glutamate transporter expression in the dorsal horn following spinal cord injury

Alexandra Pallottie[1,2], Ayomi Ratnayake[1], Li Ni[1], Cigdem Acioglu[1], Lun Li[1,2], Ersilia Mirabelli[1,2], Robert F. Heary[1,2,+] and Stella Elkabes[1,2,+,*]

1 The Reynolds Family Spine Laboratory, New Jersey Medical School, Department of Neurological Surgery, Rutgers, The State University of New Jersey, Newark, NJ, 07103, USA
2 The School of Graduate Studies, New Jersey Medical School, Rutgers, The State University of New Jersey, Newark, NJ, 07103, USA

+these authors contributed equally to this work

*Corresponding author:
Stella Elkabes
205 South Orange Avenue
Cancer Center F 1204
Newark, NJ 07003
elkabest@njms.rutgers.edu

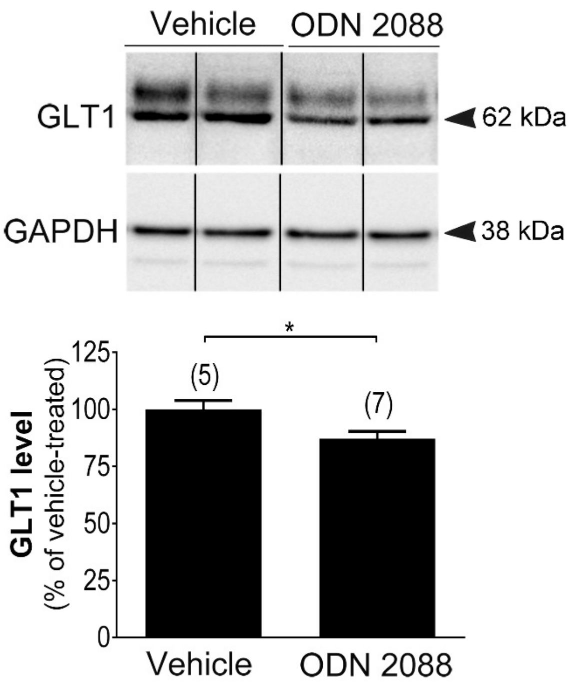

Supplementary Figure S1. Effects of ODN 2088 on GLT1 protein levels in the LDH of uninjured mice following 8 days of treatment. The upper panel shows a representative western blot (two representative lanes per treatment group). The dividing lines delineate the cropped lanes. The same exposure was applied equally across the entire image. The original pictures of the full-length western blots can be found in Supplementary Figure 10c. The lower panel is the graphic representation of the intensity of the bands after normalization to GAPDH, which was used as a control for experimental variations. Values are mean ± S.E.M. The number of mice in each group is shown in parentheses above bars. Significantly different by independent samples t-test (two-tailed), *p<0.05.

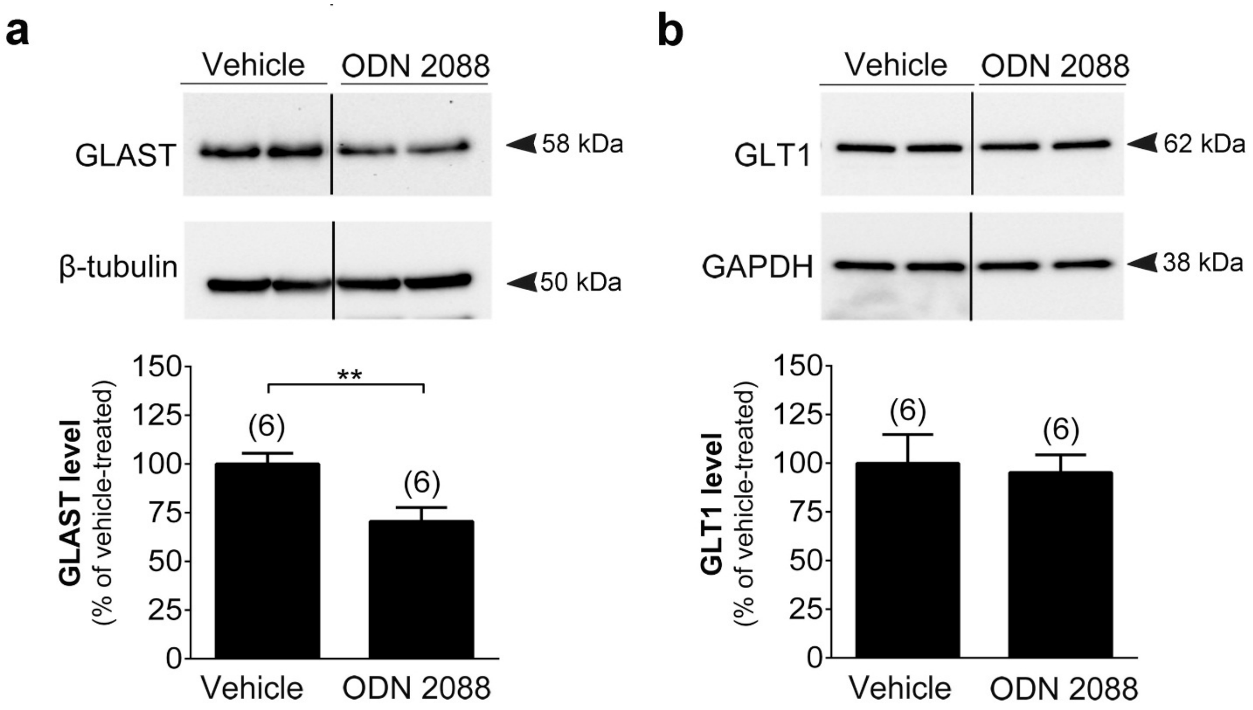

Supplementary Figure S2. Effects of ODN 2088 on GLAST and GLT1 protein levels in the LDH of uninjured mice following 28 days of treatment. (a) GLAST and (b) GLT1 protein levels. The upper panels show representative western blots (two representative lanes per treatment group). Dividing lines delineate the cropped lanes. The same exposure was applied equally across the entire image. The original pictures of the full-length western blots are shown in Supplementary Figure 10d. The lower panels are the graphic representation of the intensity of the bands after normalization to GAPDH or β-tubulin, which were used as controls for experimental variations. Values represent mean ± S.E.M. The number of mice in each group is shown in parentheses above bars. Significantly different by independent samples t-test (two-tailed), $^{**}p<0.01$.

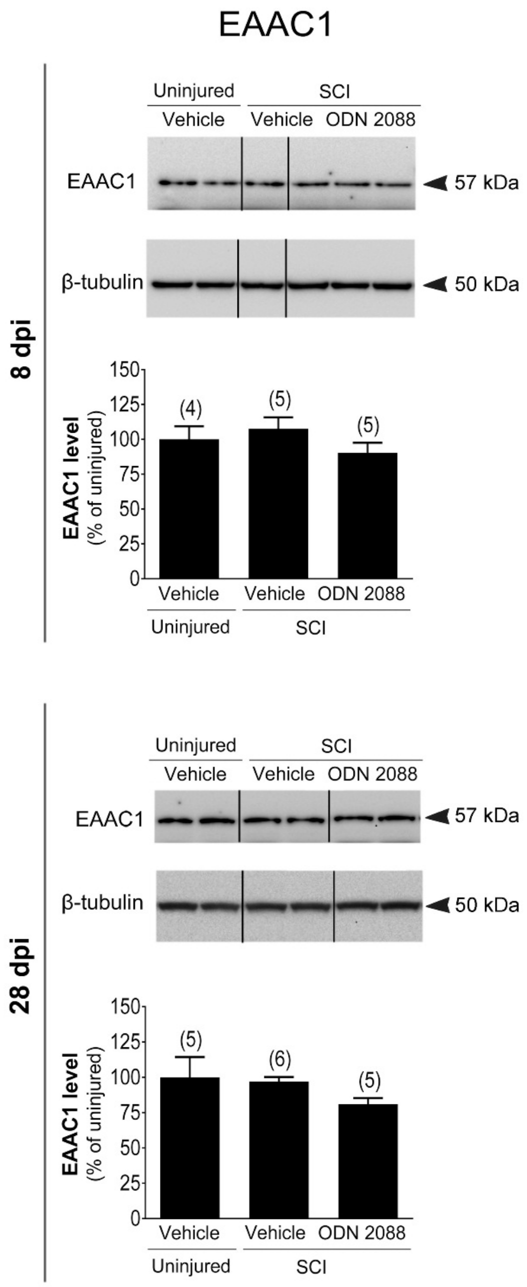

Supplementary Figure S3. Effects of ODN 2088 on EAAC1 protein expression in the LDH following a mid-thoracic SCI. The upper panels show representative western blots (two representative lanes per treatment group). Dividing lines delineate the cropped lanes. The same exposure was applied equally across the entire image. The original pictures of the full-length western blots can be found in Supplementary Figure 10e. The lower panels are the graphic representation of the intensity of the bands after normalization to β-tubulin, which was used as a control for experimental variations. Values represent mean ± S.E.M. The number of mice in each group is shown in parentheses above bars. No significant differences by one-way ANOVA.

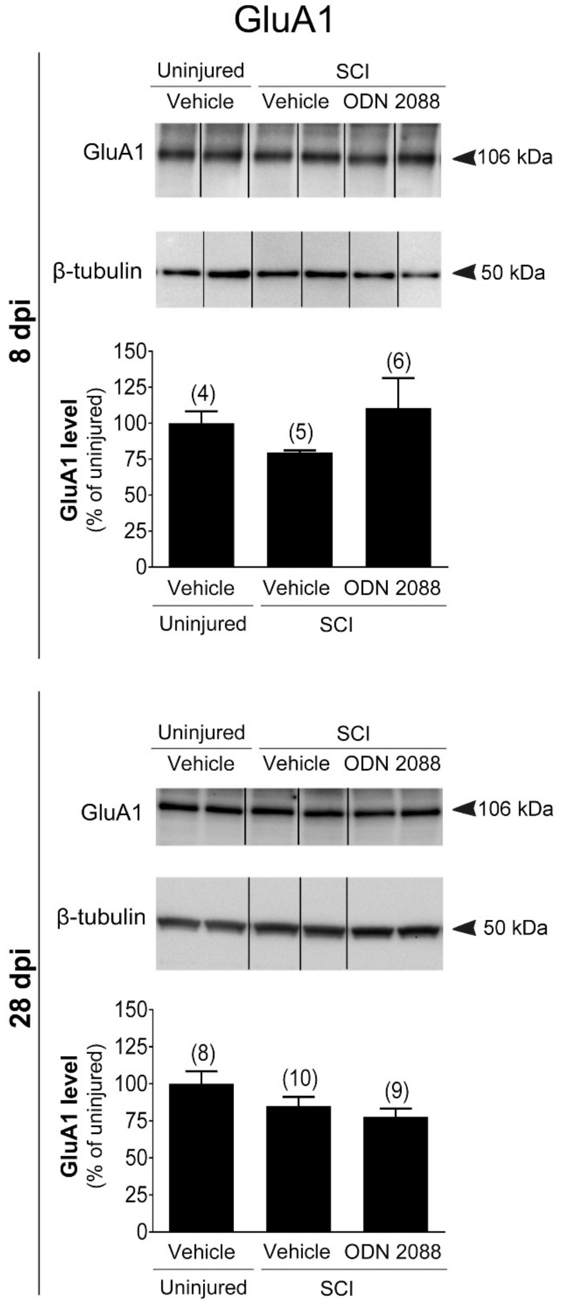

Supplementary Figure S4. Effects of ODN 2088 on GluA1 protein levels in the LDH following a mid-thoracic SCI. The upper panels show two representative lanes per treatment group, cropped from the same western blot and delineated with dividing lines. The same exposure was applied equally across the entire image. The original, full-length pictures of the western blots presented in this figure can be found in Supplementary Figure 10i. The lower panels are the graphic representation of the intensity of the bands after normalization to β-tubulin, which was used as a control for experimental variations. Values represent mean ± S.E.M. The number of mice in each group is shown in parenthesis above bars. No significant differences by one-way ANOVA.

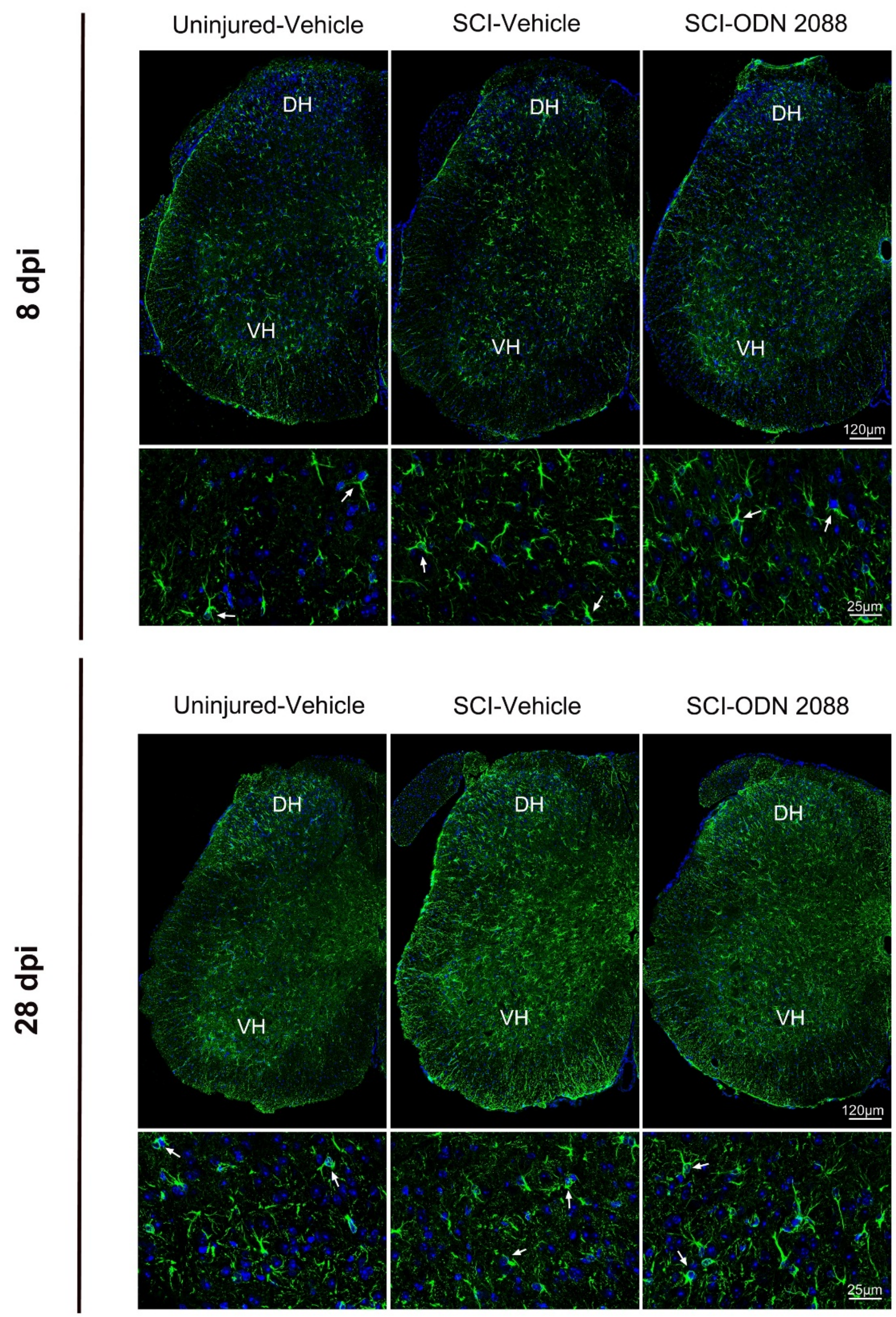

Supplementary Figure S5. GFAP-immunoreactive cells in the LDH at 8 and 28 days post-injury following treatment with vehicle or ODN 2088. Representative composite confocal images of transverse lumbar spinal cord hemi-sections immunolabeled with an antibody against GFAP (green). Nuclei have been visualized by use of DAPI (blue). Arrows point at examples of GFAP immunoreactive cells. DH: Dorsal horn, VH: Ventral horn.

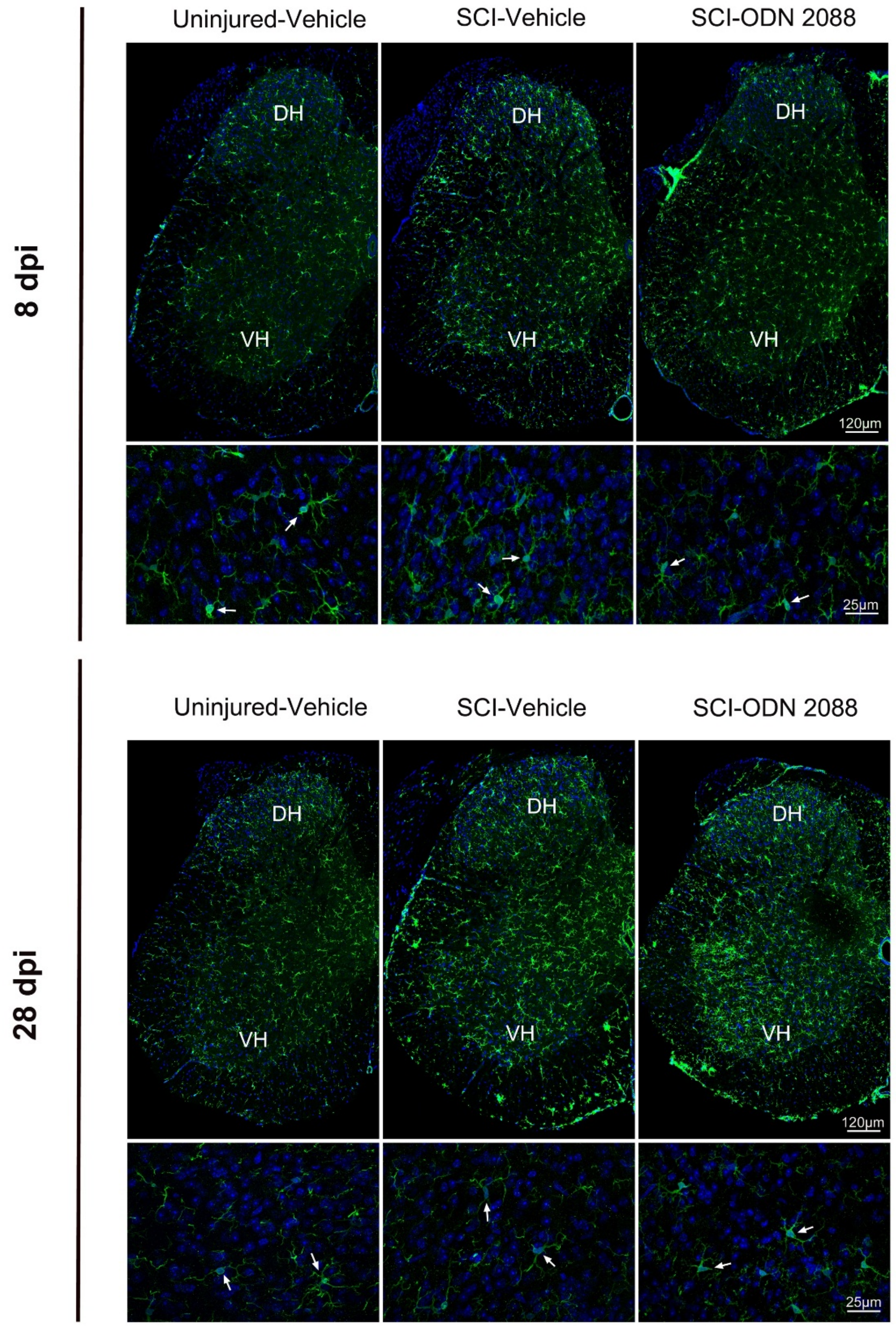

Supplementary Figure S6. Iba-1 immunoreactive cells in the LDH at 8 and 28 days post-injury following treatment with vehicle or ODN 2088. Representative composite confocal images of transverse lumbar spinal cord hemi-sections immunolabeled with an antibody against Iba-1 (green). Nuclei have been visualized by use of DAPI (blue). Arrows point at examples of Iba-1 immunoreactive cells. DH: Dorsal horn, VH: Ventral horn

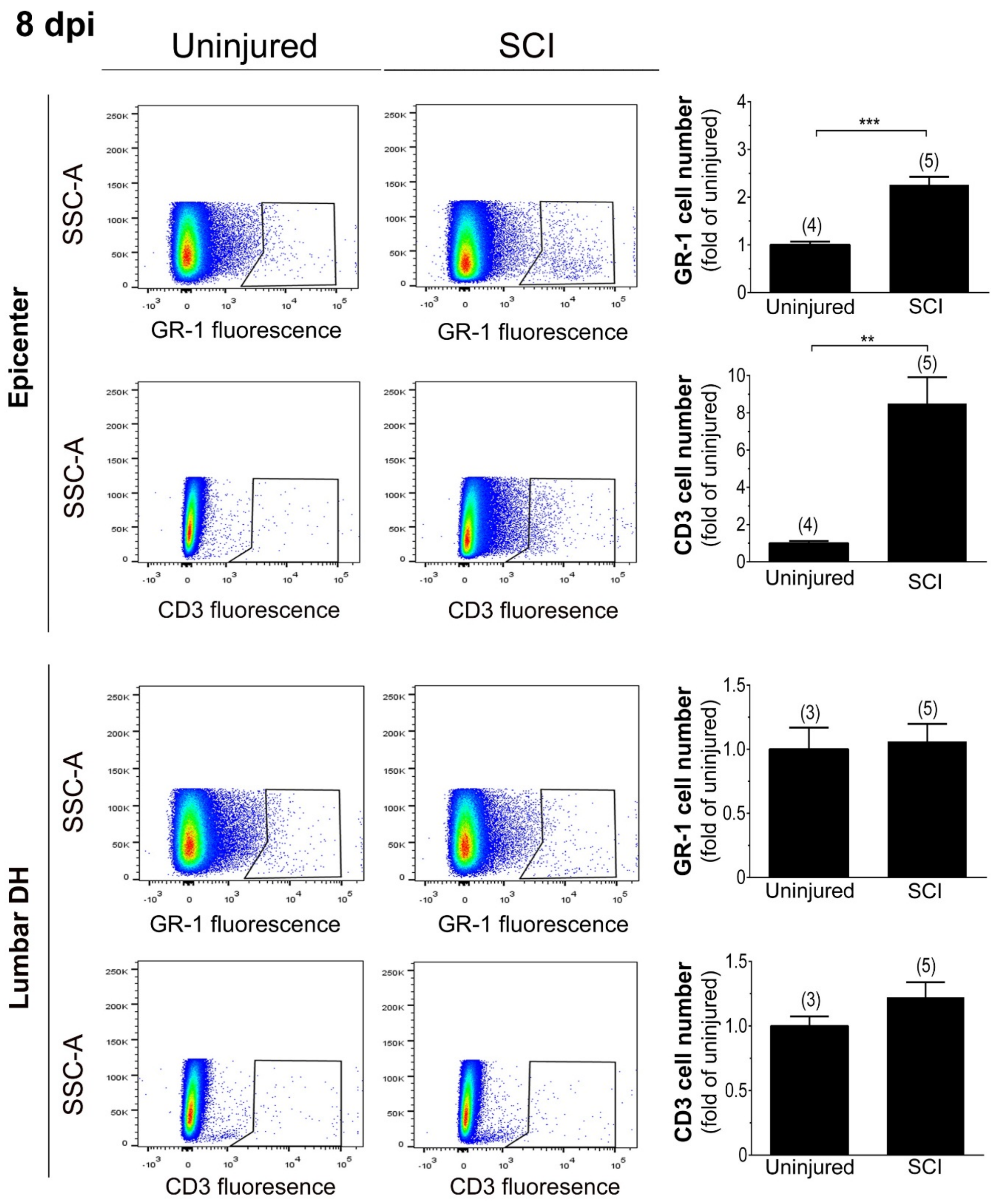

Supplementary Figure S7. GR-1+ and CD3+ cell populations in the epicenter and LDH at 8 days post-SCI. GR-1+ and CD3+ cell numbers were assessed by flow cytometry. GR-1+ and CD3+ cell populations are delineated within gates which were established based on their respective isotype controls. Values in the graphs represent mean ± S.E.M. The number of mice in each group is shown in parentheses above bars. Significant differences by independent samples t-test (two-tailed), **p<0.01, ***p<0.001

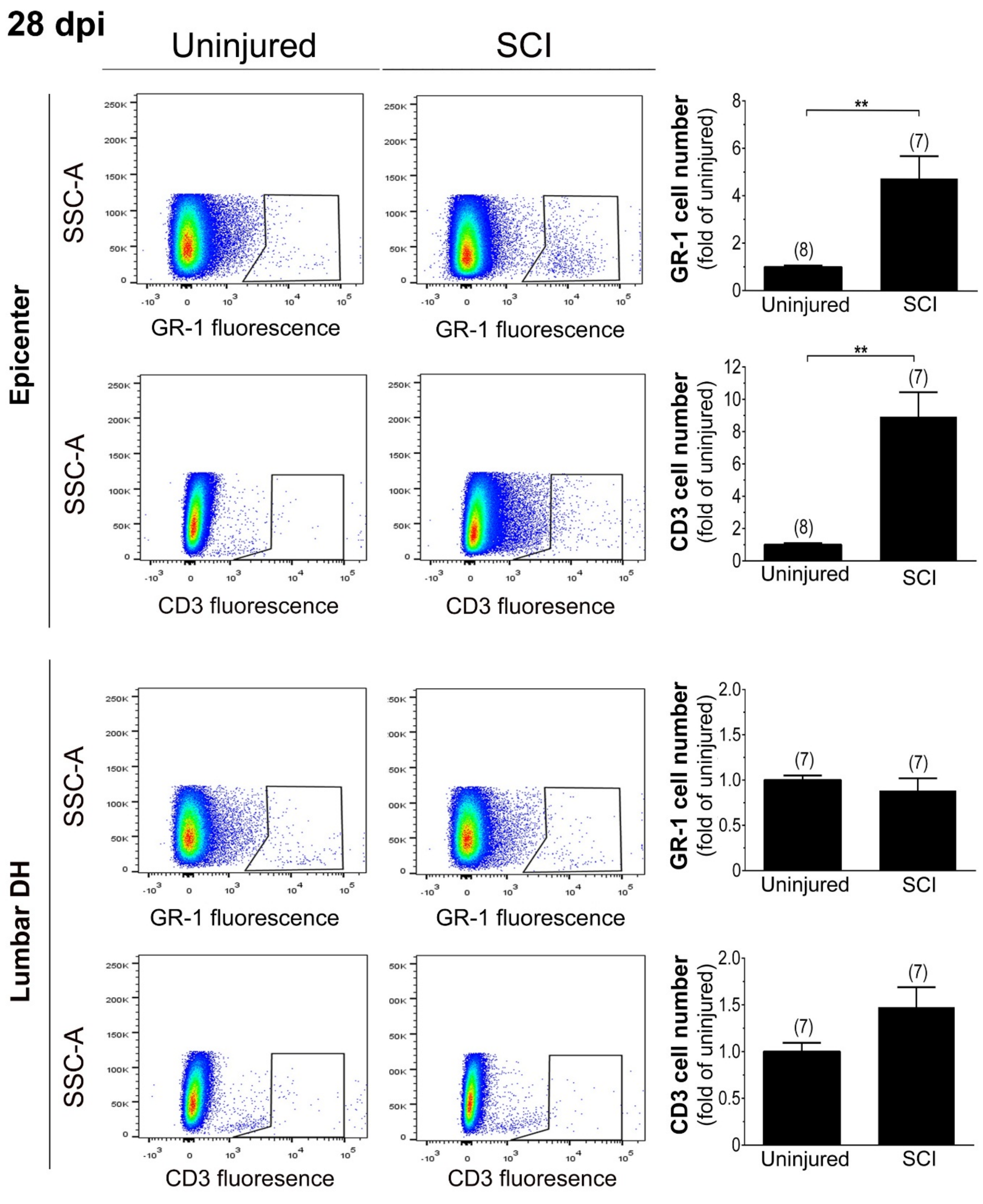

Supplementary Figure S8. GR-1+ and CD3+ cells in the epicenter and LDH at 28 days post-SCI. GR-1+ and CD3+ cell numbers were assessed by flow cytometry. GR-1+ and CD3+ cell populations are delineated within gates which were established based on their respective isotype controls. Values in the graphs represent mean ± S.E.M. The number of mice in each group is shown in parentheses above bars. Significant differences by independent samples t-test (two-tailed), **p<0.01.

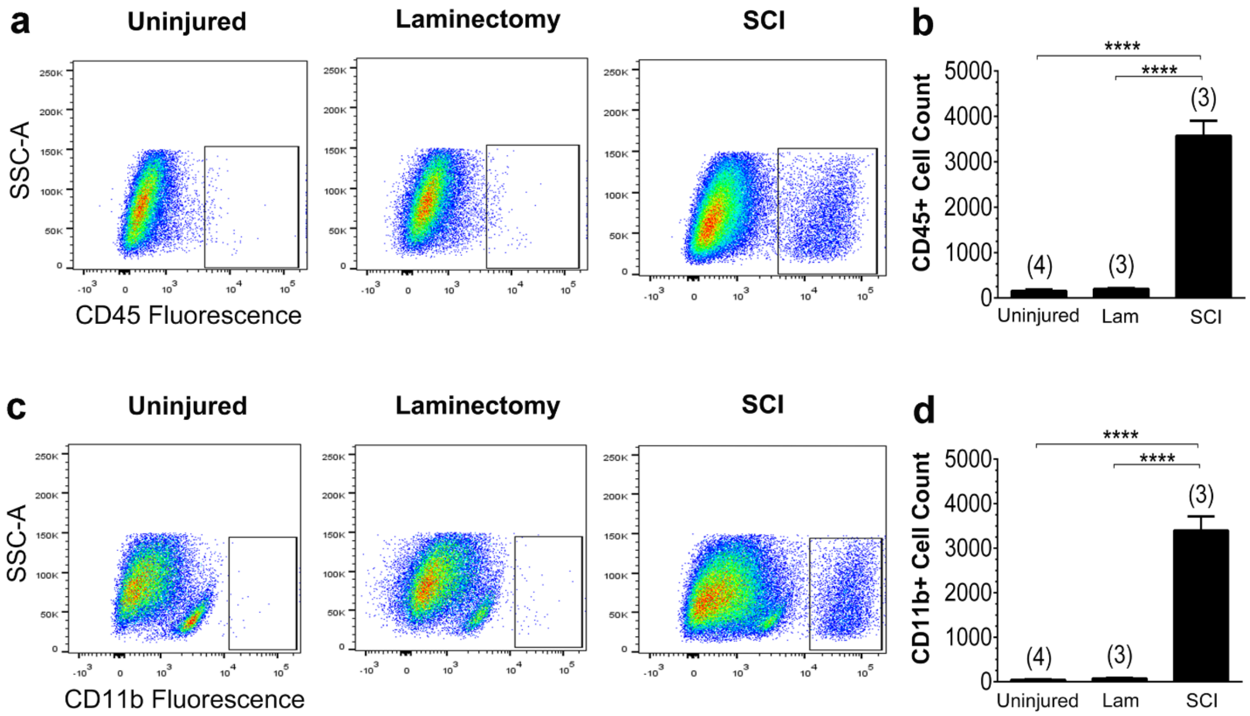

Supplementary Figure S9. CD45+ and CD11b+ cell numbers in uninjured, laminectomized and SCI-sustaining mice at thoracic T8 level. The left panels are scatterplots showing (a) $CD45^+$ and (c) $CD11b^+$ cells while the right panel is the graphic representation of (b) $CD45^+$ and (d) $CD11b^+$ cell number. CD45+ and CD11b+ cell populations are delineated within gates which were established based on their respective isotype controls. One million events were collected for each sample. Values in the graphs represent mean ± S.E.M. The number of mice in each group is shown in parentheses above bars. Significantly different by one-way ANOVA with Tukey post-hoc test, ****p<0.0001.

Supplementary Figure S10. Unaltered, full-length western blots and/or gels.

a.

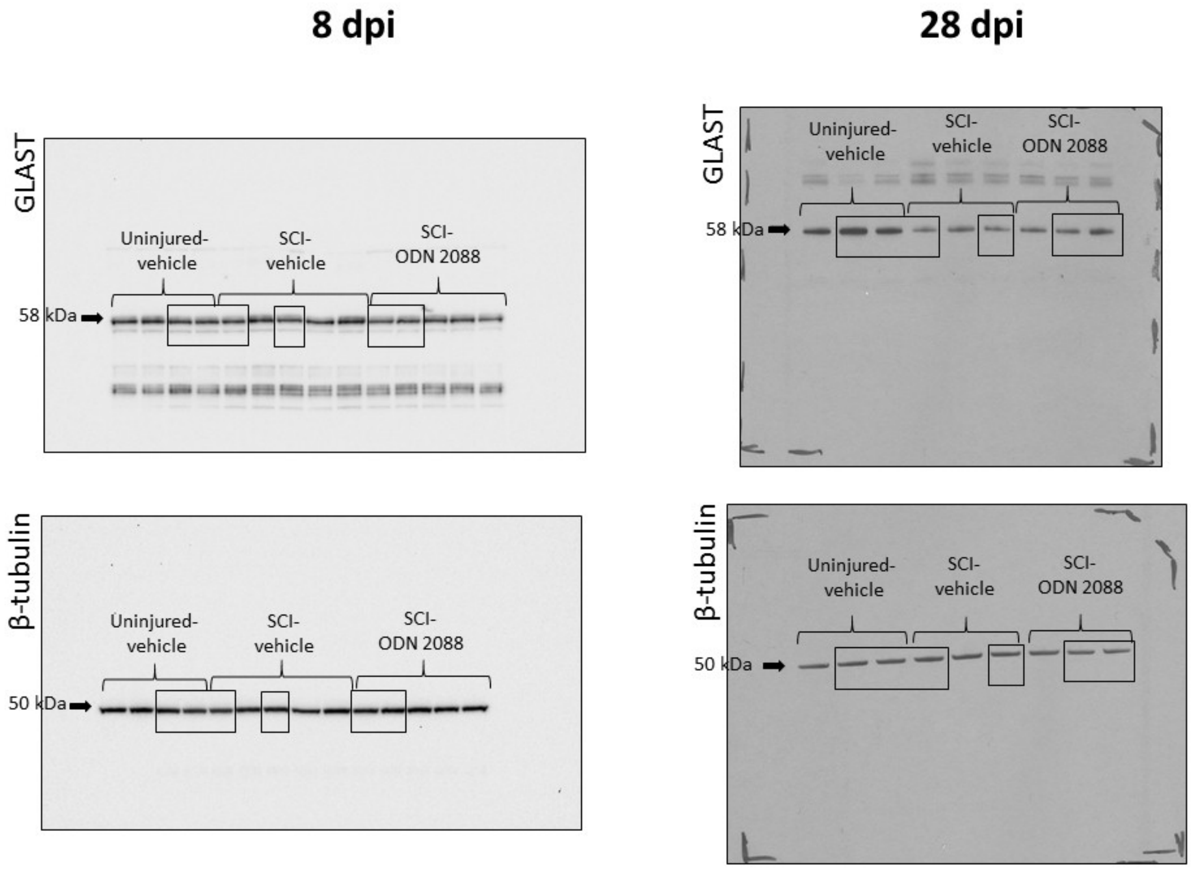

b.

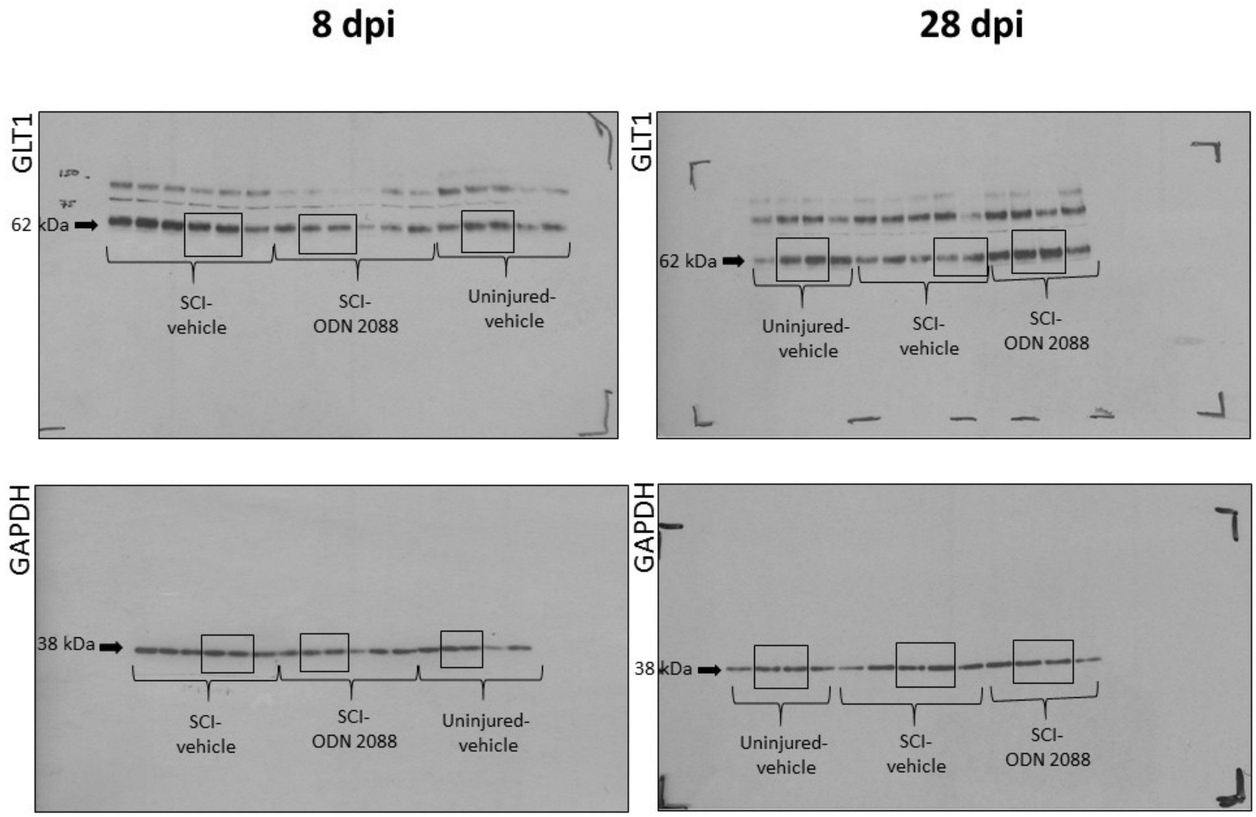
8 dpi
28 dpi
GLT1
62 kDa
SCI-
vehicle
SCI-
ODN 2088
Uninjured-
vehicle
GLT1
62 kDa
Uninjured-
vehicle
SCI-
vehicle
SCI-
ODN 2088
GAPDH
38 kDa
SCI-
vehicle
SCI-
ODN 2088
Uninjured-
vehicle
GAPDH
38 kDa
Uninjured-
vehicle
SCI-
vehicle
SCI-
ODN 2088

c.

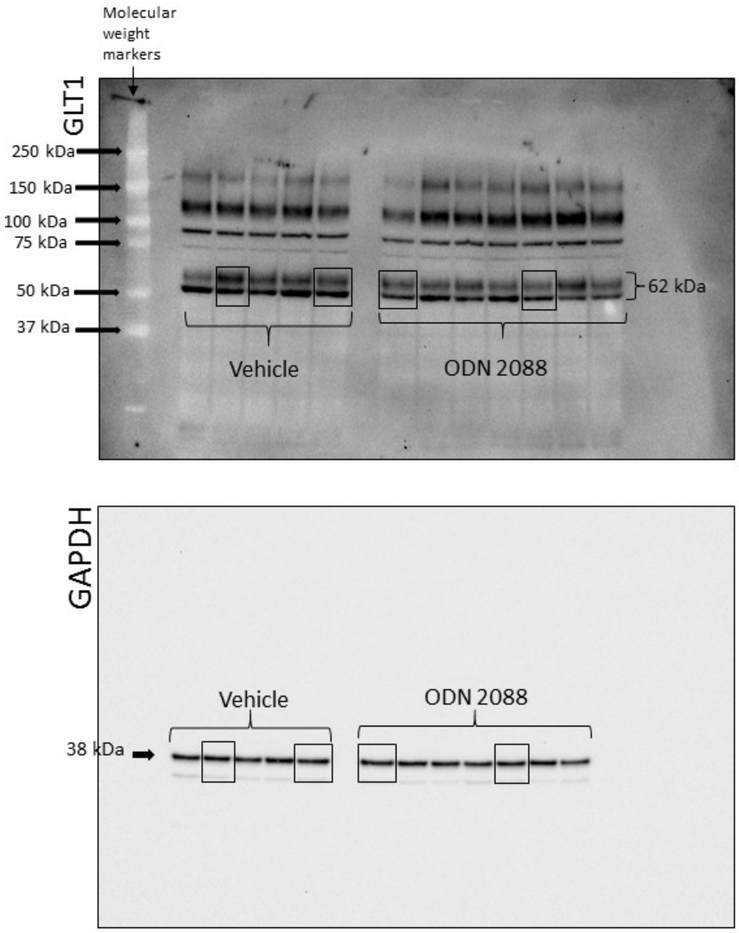
Molecular weight markers
GLT1
250 kDa
150 kDa
100 kDa
75 kDa
50 kDa
37 kDa
62 kDa
Vehicle
ODN 2088
GAPDH
Vehicle
ODN 2088
38 kDa

d.

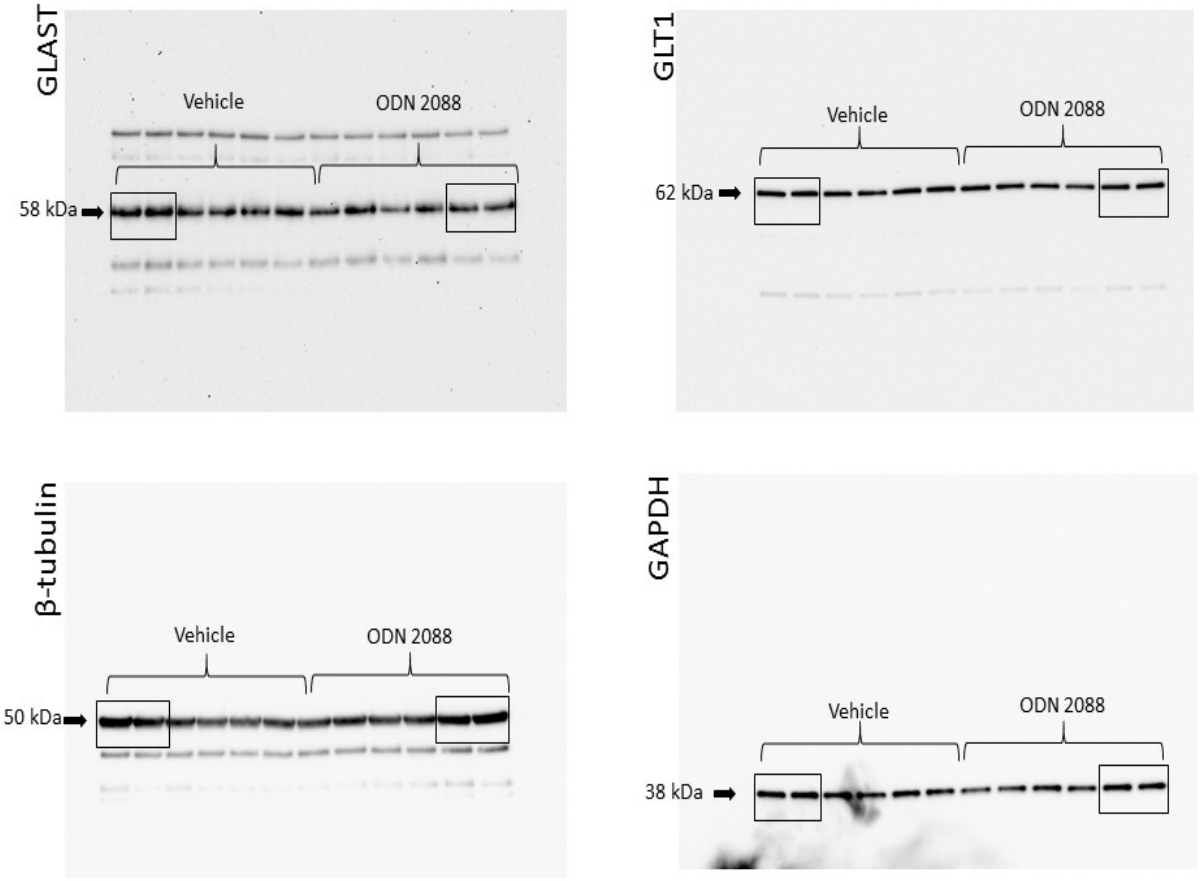
GLAST
Vehicle
ODN 2088
58 kDa
GLT1
Vehicle
ODN 2088
62 kDa
β-tubulin
Vehicle
ODN 2088
50 kDa
GAPDH
Vehicle
ODN 2088
38 kDa

e.

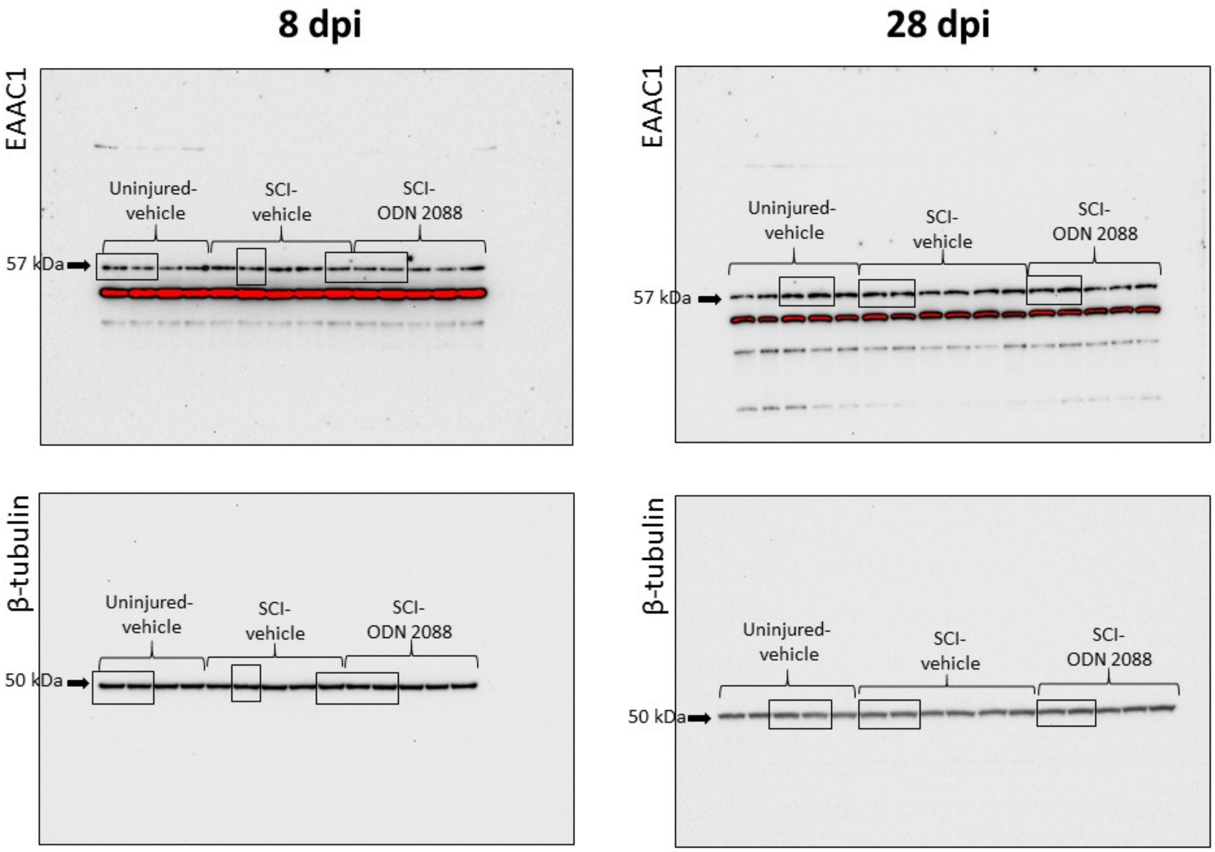
8 dpi
28 dpi
EAAC1
Uninjured-vehicle
SCI-vehicle
SCI-ODN 2088
57 kDa
β-tubulin
50 kDa

f.

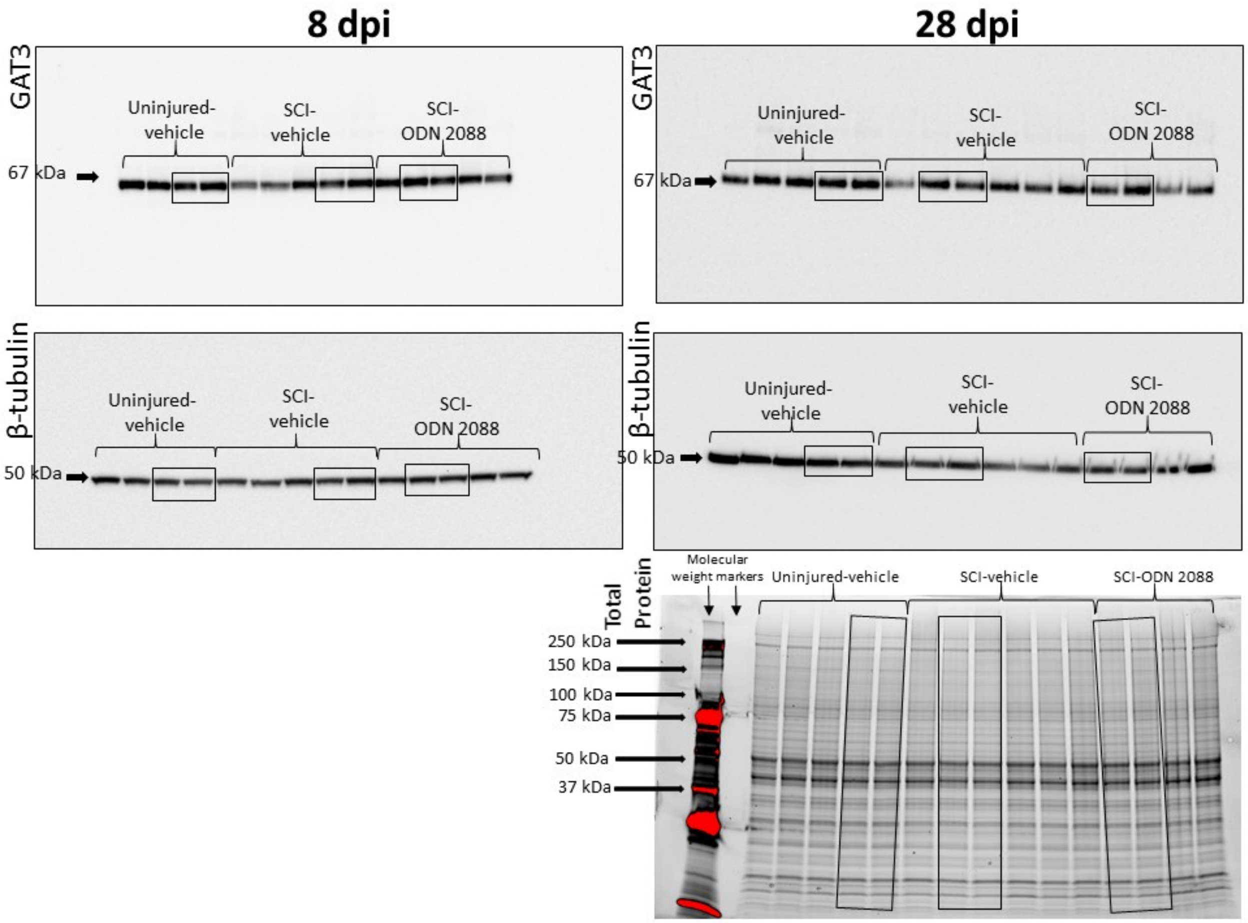
8 dpi
28 dpi
GAT3
Uninjured-vehicle
SCI-vehicle
SCI-ODN 2088
67 kDa
β-tubulin
50 kDa
Total Protein
Molecular weight markers
250 kDa
150 kDa
100 kDa
75 kDa
50 kDa
37 kDa

g.

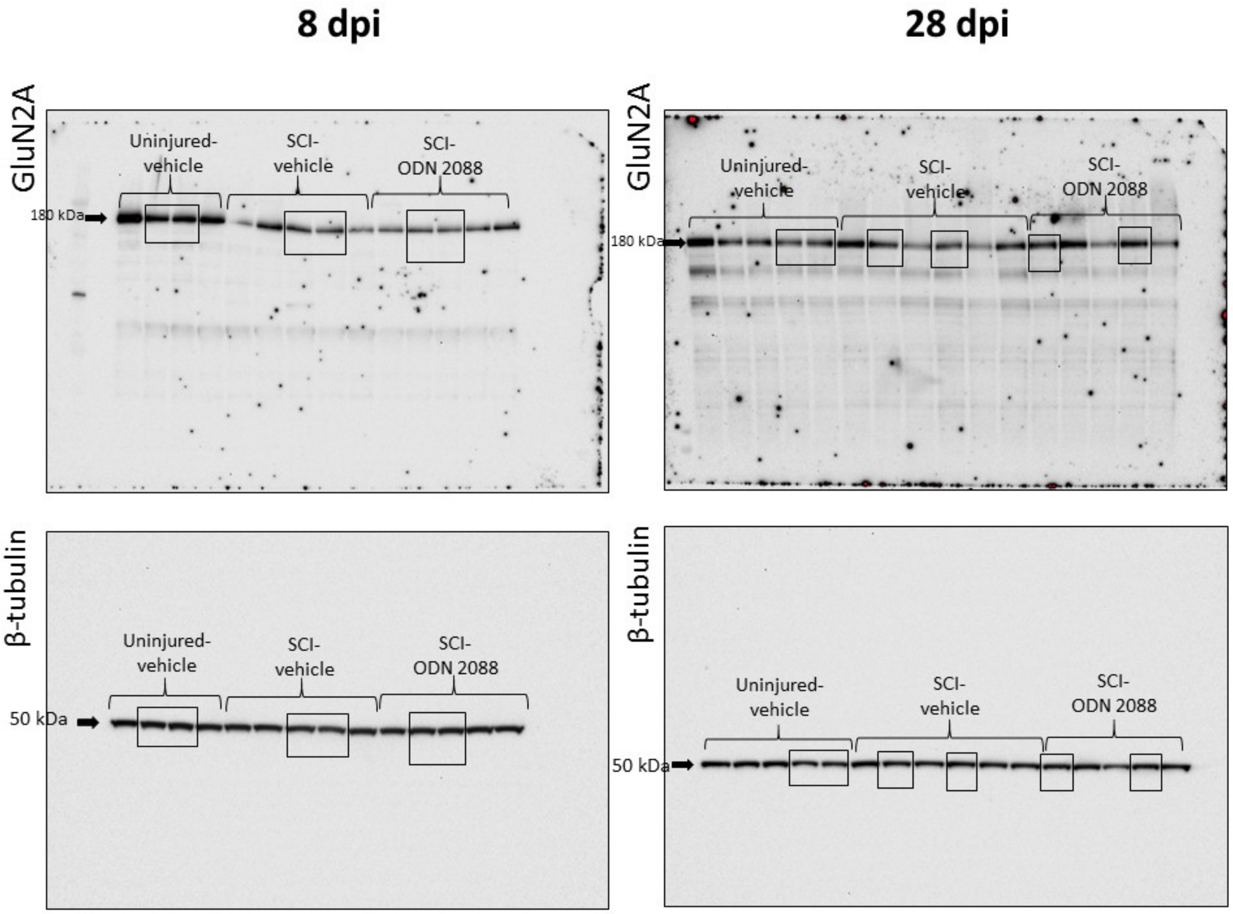

8 dpi
28 dpi
GluN2A
Uninjured-
vehicle
SCI-
vehicle
SCI-
ODN 2088
180 kDa
β-tubulin
50 kDa

h.

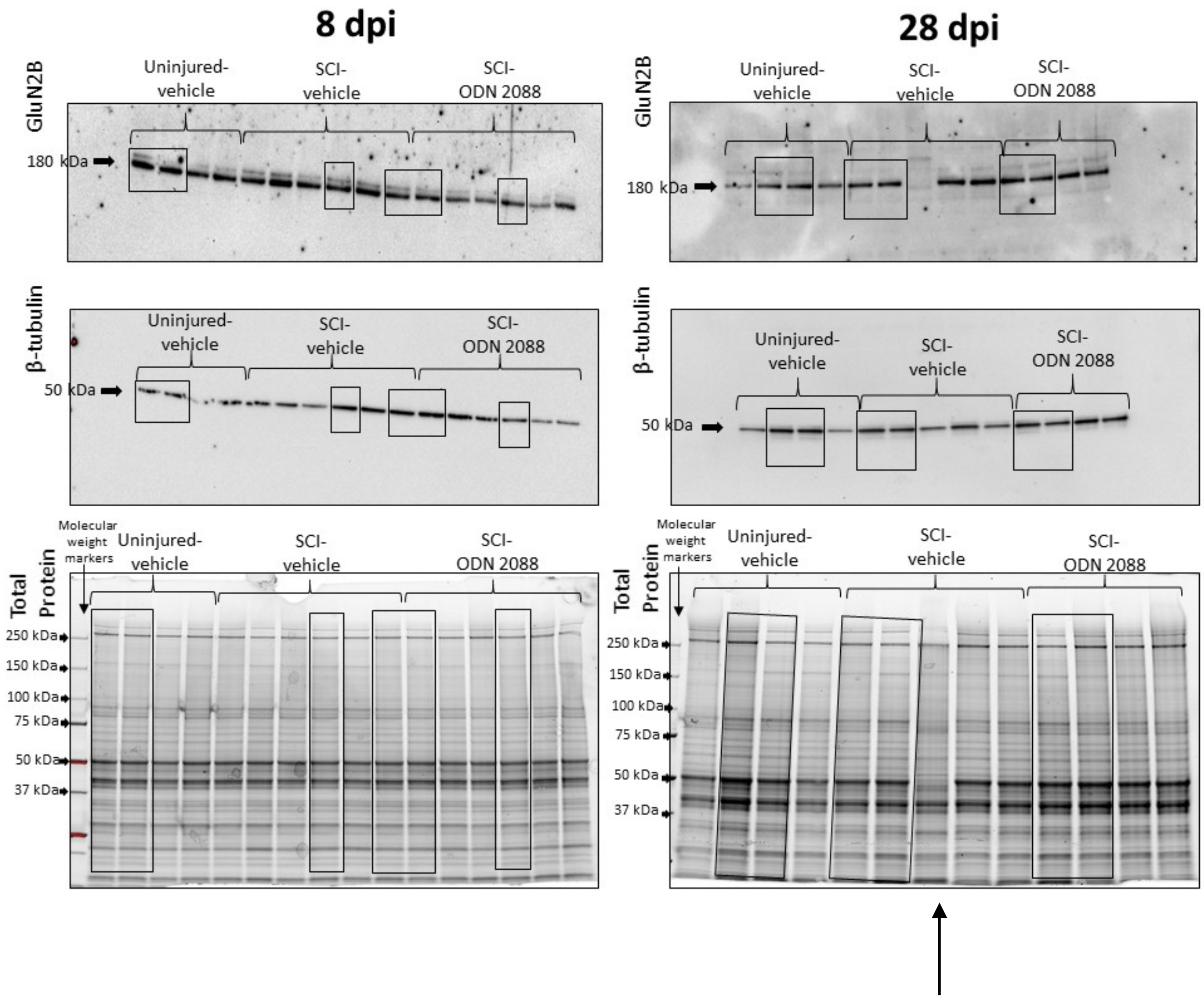

This lane was excluded from the calculations because the protein profile looked different than the rest of the lanes, suggesting some technical problem in the preparation of the sample.

i.

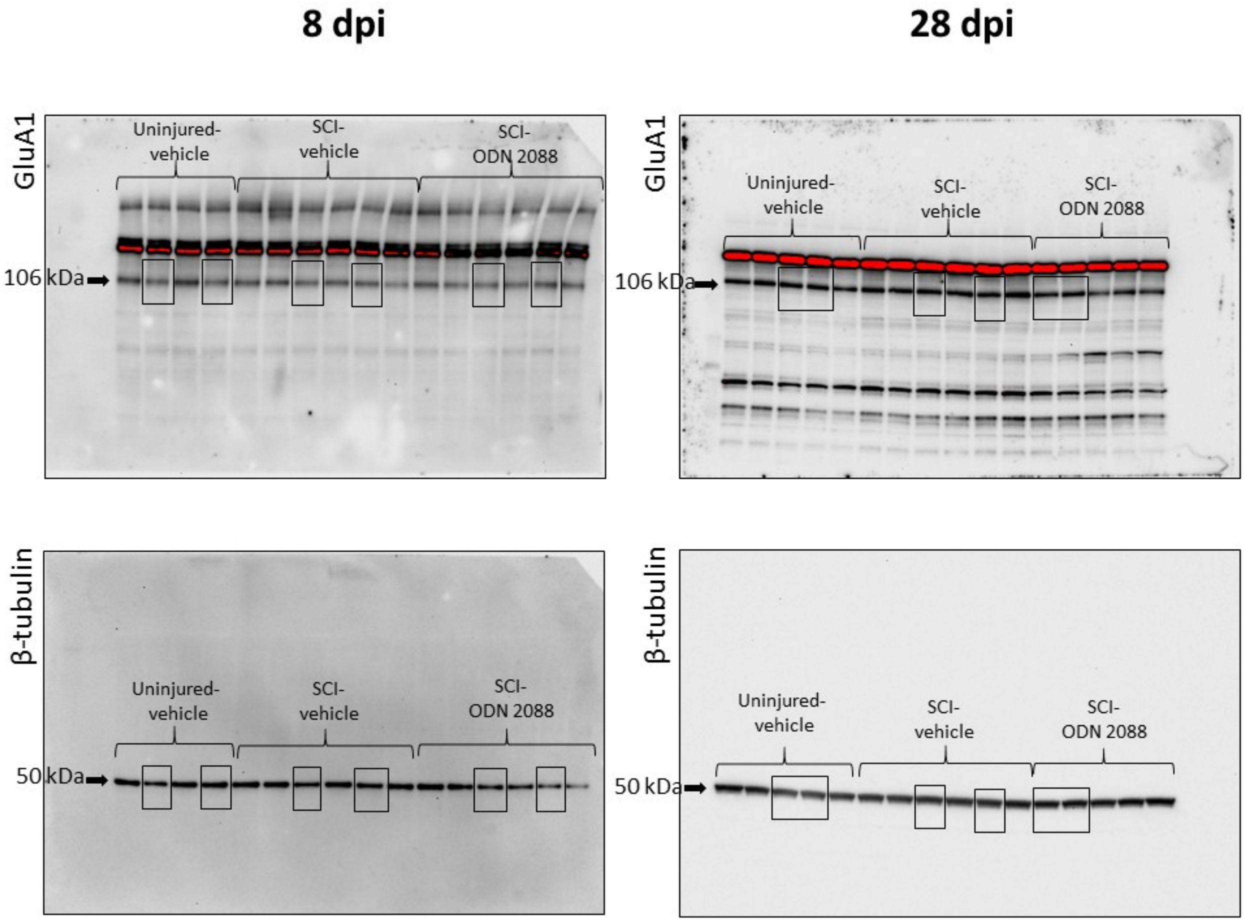
8 dpi
28 dpi
GluA1
Uninjured-vehicle
SCI-vehicle
SCI-ODN 2088
106 kDa
β-tubulin
50 kDa

j.

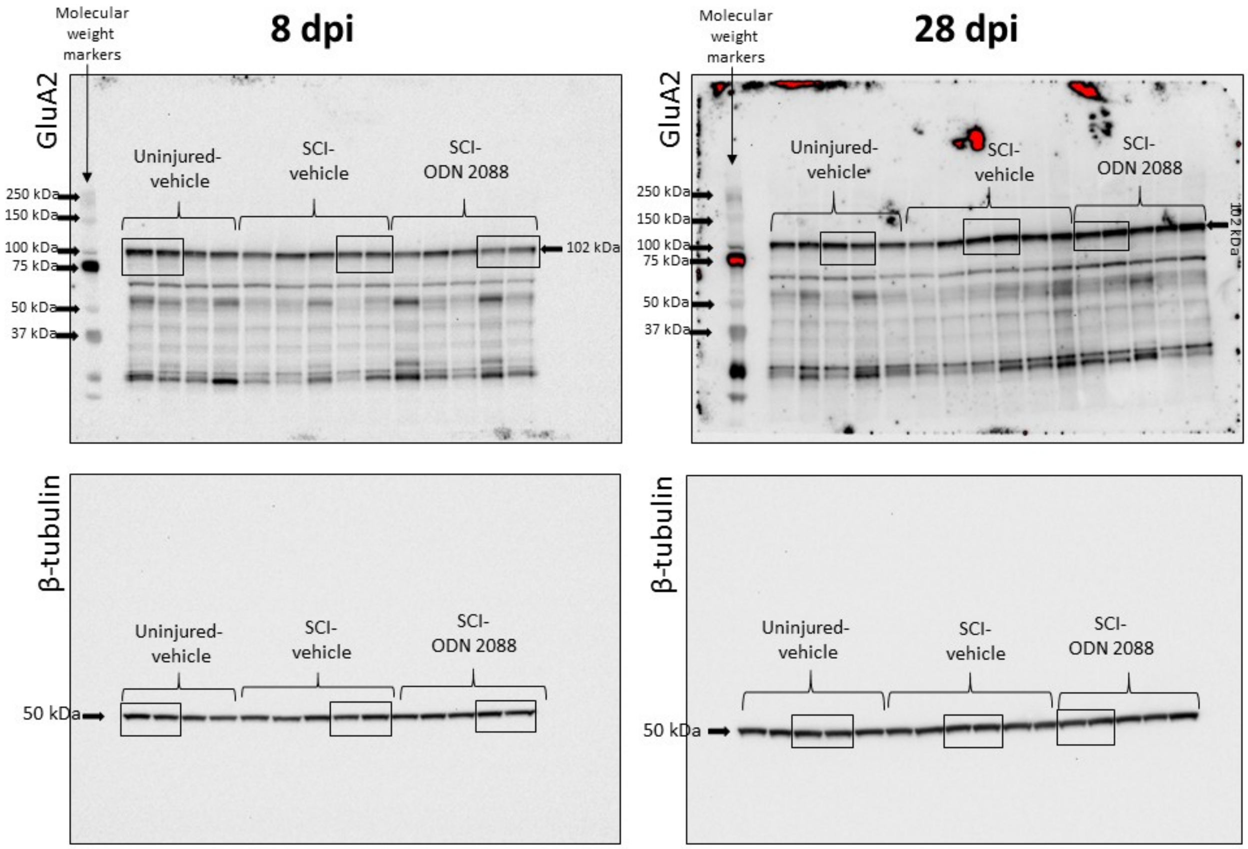
8 dpi
28 dpi
Molecular weight markers
GluA2
β-tubulin
Uninjured-vehicle
SCI-vehicle
SCI-ODN 2088
250 kDa
150 kDa
100 kDa
75 kDa
50 kDa
37 kDa
102 kDa

k.

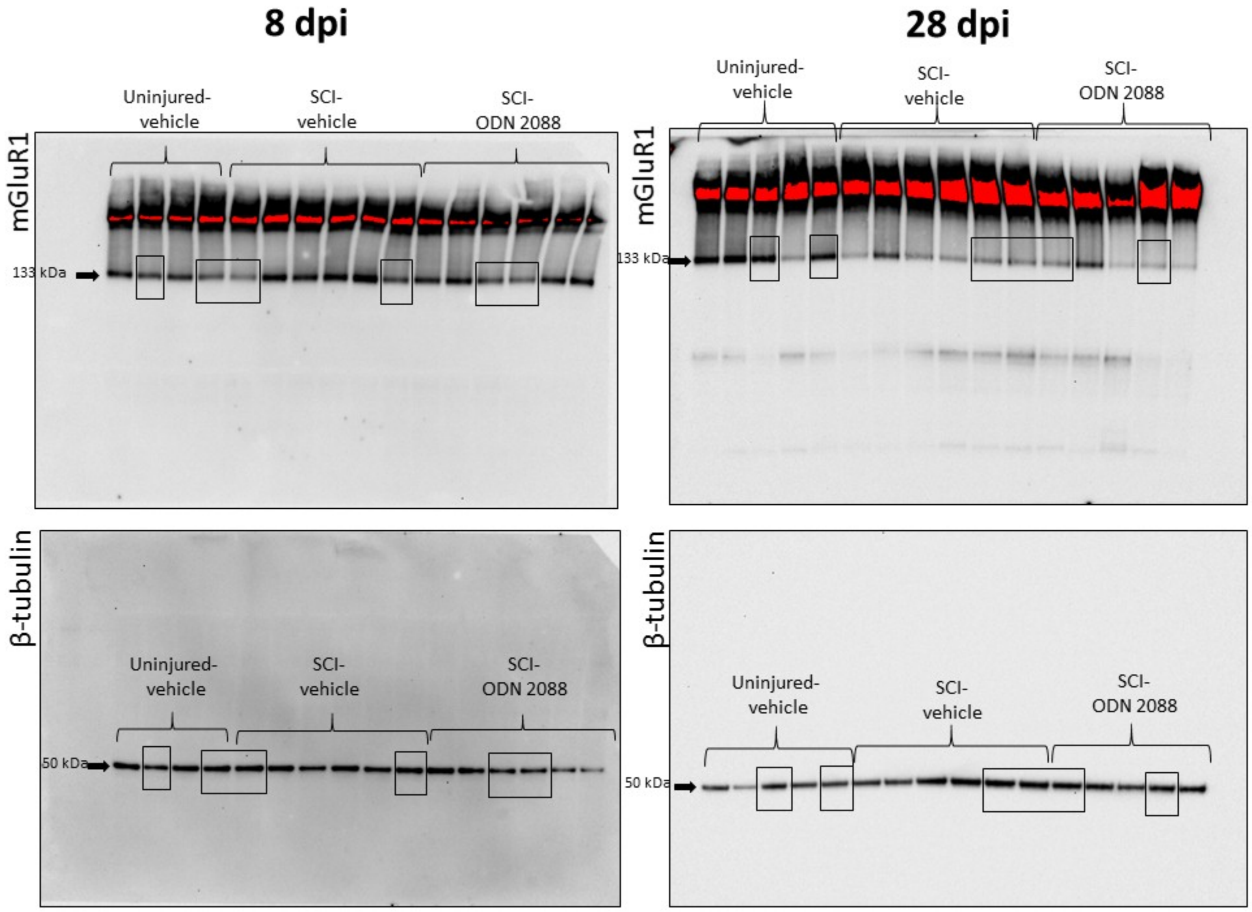
8 dpi
28 dpi
mGluR1
Uninjured-vehicle
SCI-vehicle
SCI-ODN 2088
133 kDa
β-tubulin
50 kDa

I.

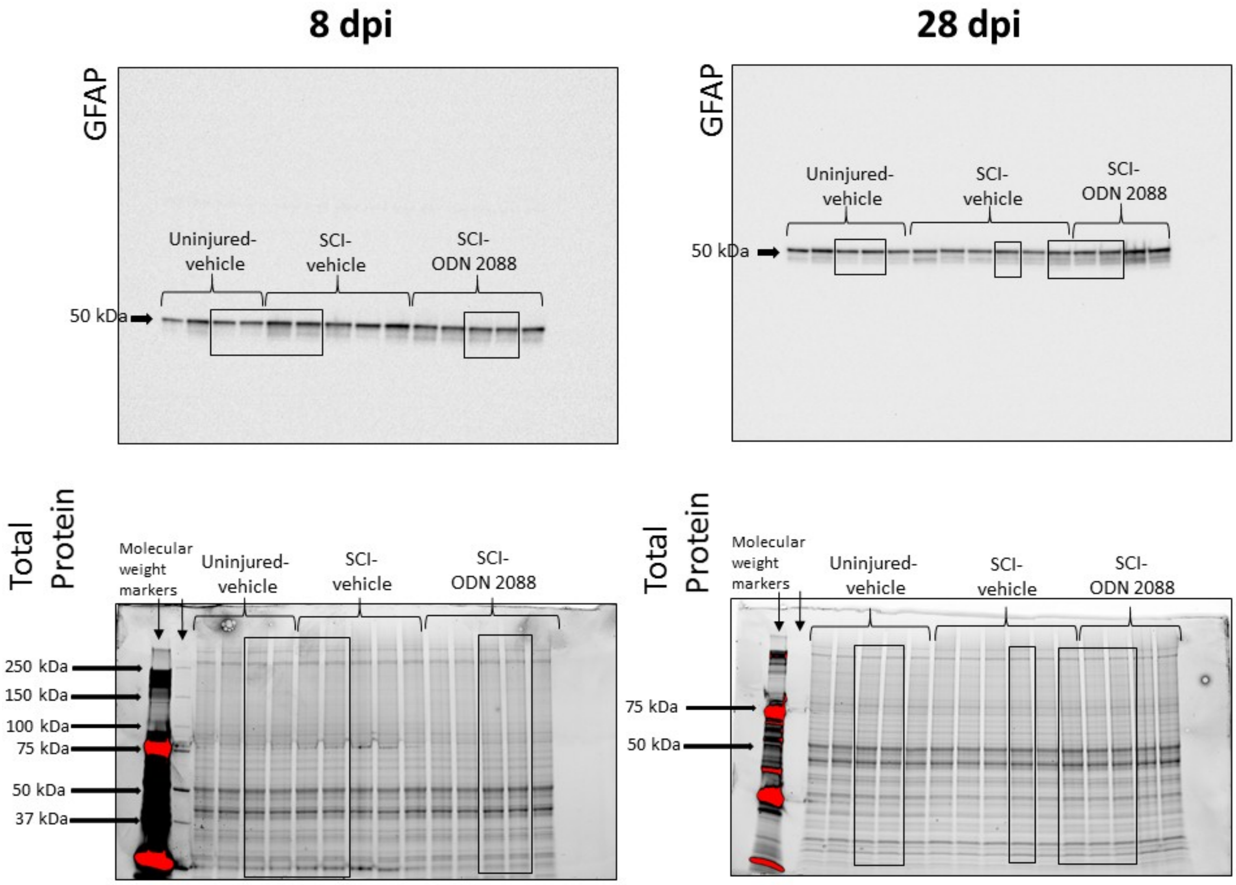
8 dpi
28 dpi
GFAP
Uninjured-
vehicle
SCI-
vehicle
SCI-
ODN 2088
50 kDa
Total
Protein
Molecular
weight
markers
250 kDa
150 kDa
100 kDa
75 kDa
50 kDa
37 kDa
